# Inhibition of MCL1 induces apoptosis in anaplastic large cell lymphoma and in primary effusion lymphoma

**DOI:** 10.1038/s41598-022-04916-6

**Published:** 2022-01-20

**Authors:** Hilmar Quentmeier, Robert Geffers, Vivien Hauer, Stefan Nagel, Claudia Pommerenke, Cord C. Uphoff, Margarete Zaborski, Hans G. Drexler

**Affiliations:** 1 Department of Human and Animal Cell Lines, Leibniz-Institute DSMZ-German Collection of Microorganisms and Cell Cultures, Inhoffenstr. 7B, 38124 Braunschweig, Germany; 2 Genome Analysis Research Group, Helmholtz Centre for Infection Research, Braunschweig, Germany

**Keywords:** Cancer, Oncology, Pathogenesis

## Abstract

Overexpression of antiapoptotic BCL2 family proteins occurs in various hematologic malignancies and contributes to tumorigenesis by inhibiting the apoptotic machinery of the cells. Antagonizing BH3 mimetics provide an option for medication, with venetoclax as the first drug applied for chronic lymphocytic leukemia and for acute myeloid leukemia. To find additional hematologic entities with ectopic expression of BCL2 family members, we performed expression screening of cell lines applying the LL-100 panel. Anaplastic large cell lymphoma (ALCL) and primary effusion lymphoma (PEL), 2/22 entities covered by this panel, stood out by high expression of MCL1 and low expression of BCL2. The MCL1 inhibitor AZD-5991 induced apoptosis in cell lines from both malignancies, suggesting that this BH3 mimetic might be efficient as drug for these diseases. The ALCL cell lines also expressed BCLXL and BCL2A1, both contributing to survival of the cells. The combination of specific BH3 mimetics yielded synergistic effects, pointing to a novel strategy for the treatment of ALCL. The PI3K/mTOR inhibitor BEZ-235 could also efficiently be applied in combination with AZD-5991, offering an alternative to avoid thrombocytopenia which is associated with the use of BCLXL inhibitors.

## Introduction

Apoptosis or programmed cell death is a process which eliminates old cells or cells that are otherwise no longer needed or detrimental. Decisive for the fate of the individual cell is the interaction between pro- and antiapoptotic members of the BCL2 family^1^. Many forms of cancer have developed mechanisms to prevent mitochondrial apoptosis by altering the equilibrium between pro- and antiapoptotic proteins^2^. Expression levels and functional importance of individual antiapoptotic proteins are considered to be different in the different types of tumors^1^.

The aberrant expression of BCL2 and of other members of the BCL2 family is well-described in follicular lymphoma (FL), chronic lymphocytic leukemia (CLL), mantle cell lymphoma (MCL), and in acute myeloid leukemia (AML)^3,4^. Mimicking proapoptotic BH3-only proteins, BH3 mimetics antagonize the function of antiapoptotic BCL2 family proteins, providing a new opportunity for treatment of these diseases^3,4^. Antagonizing BCL2, venetoclax was the first BH3 mimetic approved for CLL and then—in combination with hypomethylating drugs—also for AML^1,5^. Various new drugs targeting BCL2 family members are currently evaluated in clinical trials^1^.

Aim of our study was to identify hematologic entities not yet in the focus of broad interest, but with a conspicuous expression pattern of antiapoptotic BCL2 family members, indicating a potential for the application of BH3 mimetics. To delineate disease-specific expression patterns of antiapoptotic genes in hematologic tumors, we screened cell lines from the leukemia-lymphoma 100 (LL-100) panel. In 2019, we had developed this panel of cell lines covering 22 entities of leukemia and lymphoma malignancies as novel platform for the development of targeted therapies^6^.

RNA-seq analysis of the LL-100 panel showed that cell lines derived from two rare lymphomas, anaplastic large cell lymphoma (ALCL) and from primary effusion lymphoma (PEL) stood out by high expression of MCL1. MCL1 had already been described as essential mediator for the survival of multiple myeloma (MM) and of AML^7–9^. BH3 mimetics targeting MCL1 are promising options for the therapy of both diseases^5,8^. We describe the effects of the MCL1 inhibitor AZD-5991 on growth and apoptosis in ALCL and PEL cell lines. Furthermore, we discuss under which circumstances the combination of AZD-5991 with other BCL2 family inhibitors might be of clinical benefit. Inhibitors against the antiapoptotic AKT pathway are an alternative for combination treatment with AZD-5991.

## Results and discussion

### Expression of MCL1 and BCL2 in cell lines of the LL-100 panel

Overexpression of antiapoptotic BCL2 family genes promotes survival of cells in hematological malignancies^10,11^. Deregulation of BCL2 in hematopoietic tumors could be traced back to the translocation t(14;18), amplification or hypomethylation of the gene^2^.

**Figure 1:**
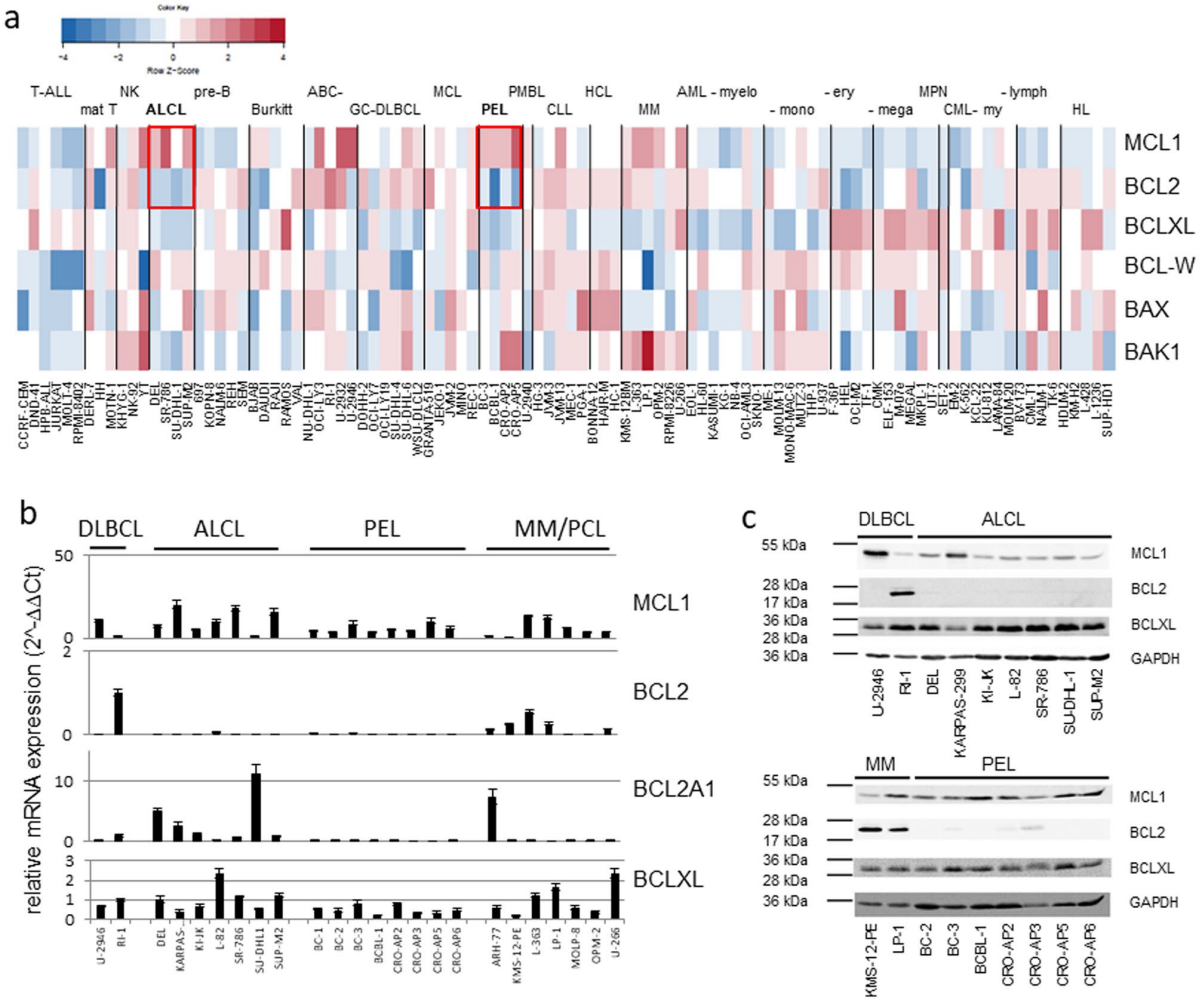
Expression of BCL2 family genes in the LL-100 panel. (**a**) Heat map of RNA-seq gene expression data of BCL2 family members. Framed in red are expression levels of MCL1 and BCL2 in ALCL and PEL. (**b**) Results of qRT-PCR showing expression of BCL2 family members. (**c**) Western blot analysis showing the protein expression of MCL1, BCL2 and BCLXL in ALCL and PEL cell lines. DLBCL and MM cell lines were included as controls. Note that ALCL and PEL cell lines are consistently MCL1^pos^/BCL2^neg^.

MCL1 is one of the eminent BCL2 family members which, when overexpressed, contributes significantly to the survival of malignant cells^7,12^. To find potential targets of BH3 mimetics, we examined the expression of BCL2 family genes in the LL-100 cell lines panel. Remarkably, all ALCL and PEL cell lines showed an MCL1^high^/BCL2^low^ expression pattern (Fig. 1a). Quantitative reverse transcriptase (qRT) PCR analysis with an extended number of cell lines confirmed expression of MCL1 and absence of BCL2 in ALCL and PEL cell lines (Fig. 1b). Western blot analysis verified this finding at the protein level (Fig. 1c).

### MCL1 amplification in ALCL and PEL cell lines

MCL1 is the dominant antiapoptotic BCL2 family member in MM, more critical than BCL2 and BCLXL^7,8^. MCL1 is also essential for survival of AML cells^5,9,13,14^ and of Philadelphia chromosome positive acute lymphocytic leukemia (ALL) cells^15^.

The *MCL1* gene is located on chromosome 1 (q21.3), in a region which is often amplified in human cancer^16^. According to our quantitative genomic PCR, 6/7 ALCL cell lines and 6/8 PEL cell lines carry gains of *MCL1* (Suppl. Table 1). In most cell lines, the gains result from global tri- or tetraploidies or from aneuploidies of chromosome 1 (Suppl. Table 1). But the ALCL cell line with highest MCL1 protein expression levels (KARPAS-299) carries indeed a strong amplification of the *MCL1* locus (9n), suggesting that focal gain can play a role in the aberrant overexpression of *MCL1* (Fig. 1c, Suppl. Table 1).

Providing an alternative explanation for *MCL1* expression in ALCL, the cytokine-induced activation of the TYK2/STAT1 pathway triggers *MCL1* in this disease^17^.

### Effect of BH3 mimetics on PEL cell lines

MCL1 and BCL2 are antiapoptotic BCL2 family members. MCL1 is overexpressed in ALCL and PEL tumors and protects cells from apoptosis^18–20^. Figure 1c shows that cell lines from both hematologic entities are MCL1^pos^/BCL2^neg^, suggesting that they might represent disease-specific models to study the efficiency of MCL1 inhibitors.

Inhibitors of antiapoptotic BCL2 family proteins (BH3 mimetics) are emerging agents for the treatment of BCL2 dependent tumors. Different compounds are in clinical trials, with the BCL2 inhibitor Venetoclax (ABT-199) being the first BH3 mimetic approved by the FDA for CLL^1^.

Before testing ALCL and PEL cell lines, we examined the growth inhibitory effects of three MCL1 inhibitors (AT-101, AZD-5991, UMI-77) and of one BCL2/BCLXL inhibitor (ABT-263) on the diffuse large B cell lymphoma (DLBCL) cell lines U-2946 (MCL1^pos^/BCL2^neg^) and RI-1 (MCL1^low^/ BCL2^pos^) (Fig. 1c). These cell lines were chosen as controls, as we had assessed their sensitivity for BH3 mimetics in an earlier study^21^. ABT-263 inhibited growth of the BCL2^pos^ cell line (Ri-1), AZD-5991 efficiently inhibited growth of the MCL1^pos^ cell line (U-2946) (Table 1, Suppl. Fig. 1a–c). The activity of caspases 3/7 was induced accordingly, confirming that the effects of the drugs on cell counts resulted from apoptosis (Suppl. Fig. 1b,c). Two MCL-1 inhibitors (AT-101 and UMI-77) were active in the µM range (data not shown) and hence no longer used in this study.

**Table 1:**
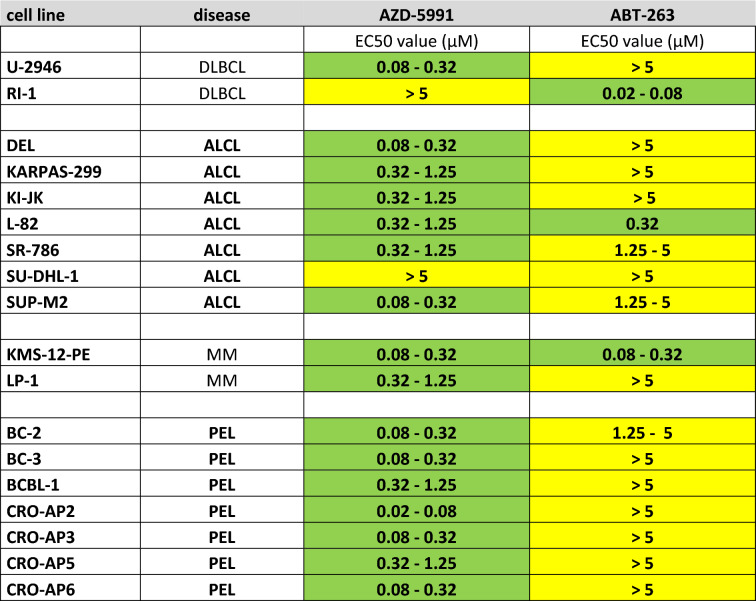
Effects of AZD-5991 and ABT-263 on growth of lymphoma cell lines.

EC50 values were determined after 48 h by life cell imaging. Green indicates inhibitor sensitivity of cell line (EC50 < 1.25 µM); yellow indicates resistance (EC50 ≥ 1.25 µM). EC50 values for BI-97C1 targeting various BCL2 family members including BCL2A1 were identical between the ALCL cell lines L-82 and SU-DHL-1 (1.25–5 µM).

Western blot analysis showed that PEL cell lines are MCL1^pos^/BCL2^neg^ (Fig. 1c). Therefore, it was not surprising that all PEL cell lines tested (7/7) were sensitive to the MCL1 inhibitor AZD-5991 (EC50 < 1.25 µM) and resistant to the BCL2 inhibitor ABT-263 (EC50 ≥ 1.25 µM) (Table 1).

PEL is a rare and aggressive non-Hodgkin lymphoma, with poor prognosis^22,23^. PEL cell lines exhibit a characteristic gene expression profile including high levels of CD138, PRDM1 and SLAMF7, the latter being a potential candidate for targeted antibody therapy^6^. We show here that the MCL1 inhibitor AZD-5991 induces apoptosis in PEL cell lines. These results confirmed the importance of MCL1 for survival of PEL^20^.

Thus, PEL patients might benefit from targeted therapy with MCL1 antagonists. Nevertheless, our cell lines data need to be confirmed in primary tumor samples.

### Effect of BH3 mimetics on ALCL cell lines

Like PEL cell lines, also ALCL cell lines are MCL1^pos^/BCL2^neg^ (Fig. 1c). However, only five out of seven ALCL cell lines were sensitive to the MCL1 inhibitor and resistant to the BCL2 inhibitor, begging the question why cell line SU-DHL-1 was resistant for both inhibitors and why cell line L-82 responded to both inhibitors (Table 1).

ABT-263 inhibits not only BCL2, but also BCLXL and BCL-W. Therefore, expression of the latter two genes might explain responsiveness of a BCL2^neg^ cell line to this drug. Western blot analysis allowed detection of BCLXL in ALCL cell lines, but showed only faint signals for BCL-W (Suppl. Fig. 2). In accordance, C_T_ values of BCL-W, as determined by qRT-PCR, were decisively higher than those of MCL1 and BCLXL (> 5 C_T_ value difference; data not shown). Therefore, we assessed BCLXL as candidate antiapoptotic mediator in ALCL.

**Figure 2:**
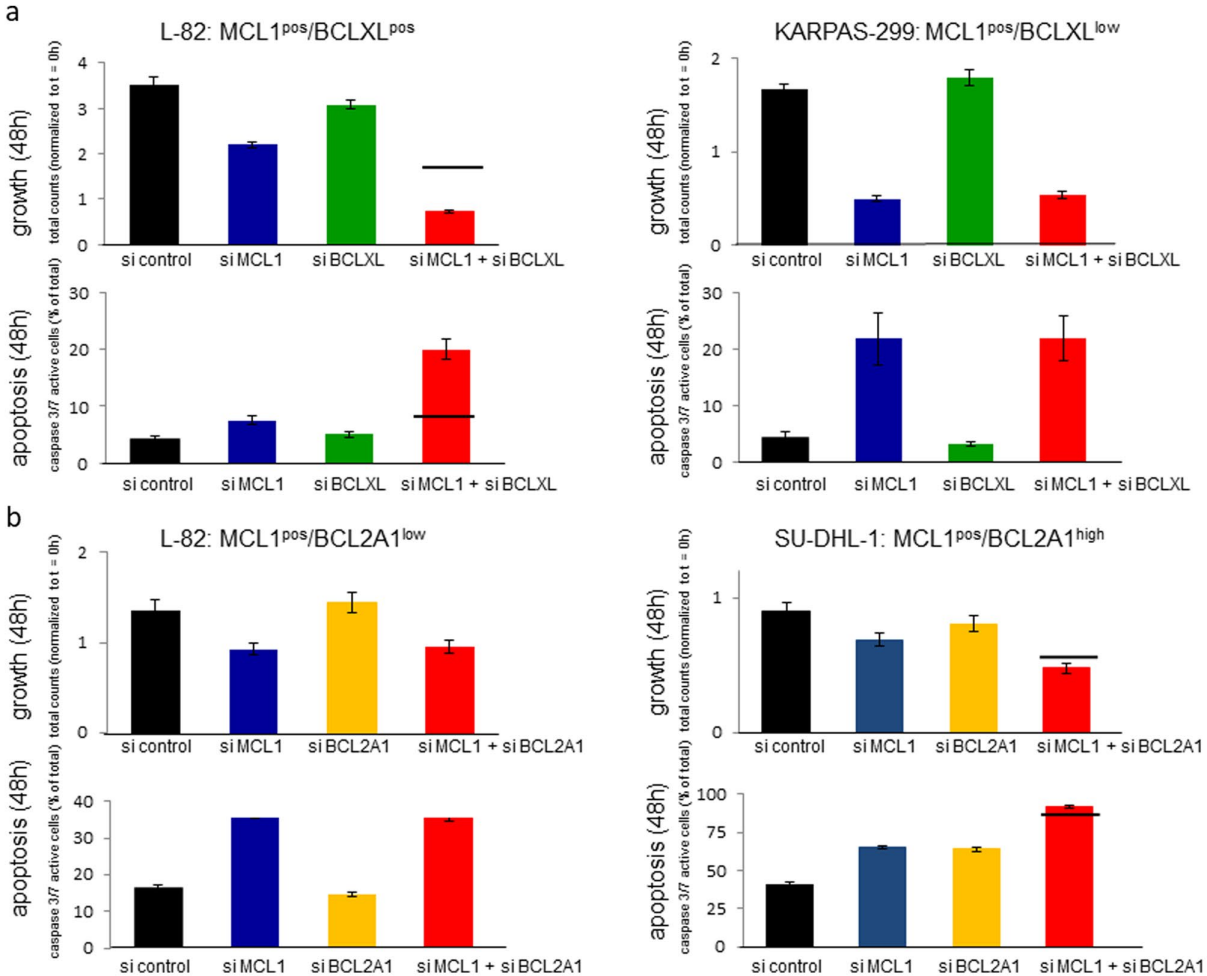
SiRNA-mediated knockdown of BCL2 family members in ALCL cell lines. Life cell imaging shows effects of knockdown of BCL2 family members on growth (cell counts) and apoptosis (caspase 3/7 activity) 48 h after onset of treatment with siRNA oligos. (**a**) Knockdown of MCL1 and BCLXL; (**b**) knockdown of MCL1 and BCL2A1. Note synergistic effects of MCL1 and BCLXL knockdown in cell line L-82 and additive effects of MCL1 and BCL2A1 knockdown in cell line SU-DHL-1. The horizontal line shows the expected additive effects of the combination of siRNA oligos.

Knock-down experiments were performed to elucidate whether BCLXL was crucial for survival of the ALCL cell lines. The efficiency of knockdown with short interfering (si) RNA oligonucleotides was verified by qRT-PCR (Suppl. Fig. 3). MCL1 and BCLXL knockdown led to the reduction of growth in the MCL1^pos^/BCLXL^pos^ cell line L-82 (Fig. 2a). Co-knockdown of both genes yielded more than additive effects with respect to growth arrest and induction of apoptosis (Fig. 2a). The MCL1^pos^/BCLXL^low^ cell line KARPAS-299 responded to knockdown of MCL1, but not to knockdown of BCLXL (Fig. 2a).

**Figure 3:**
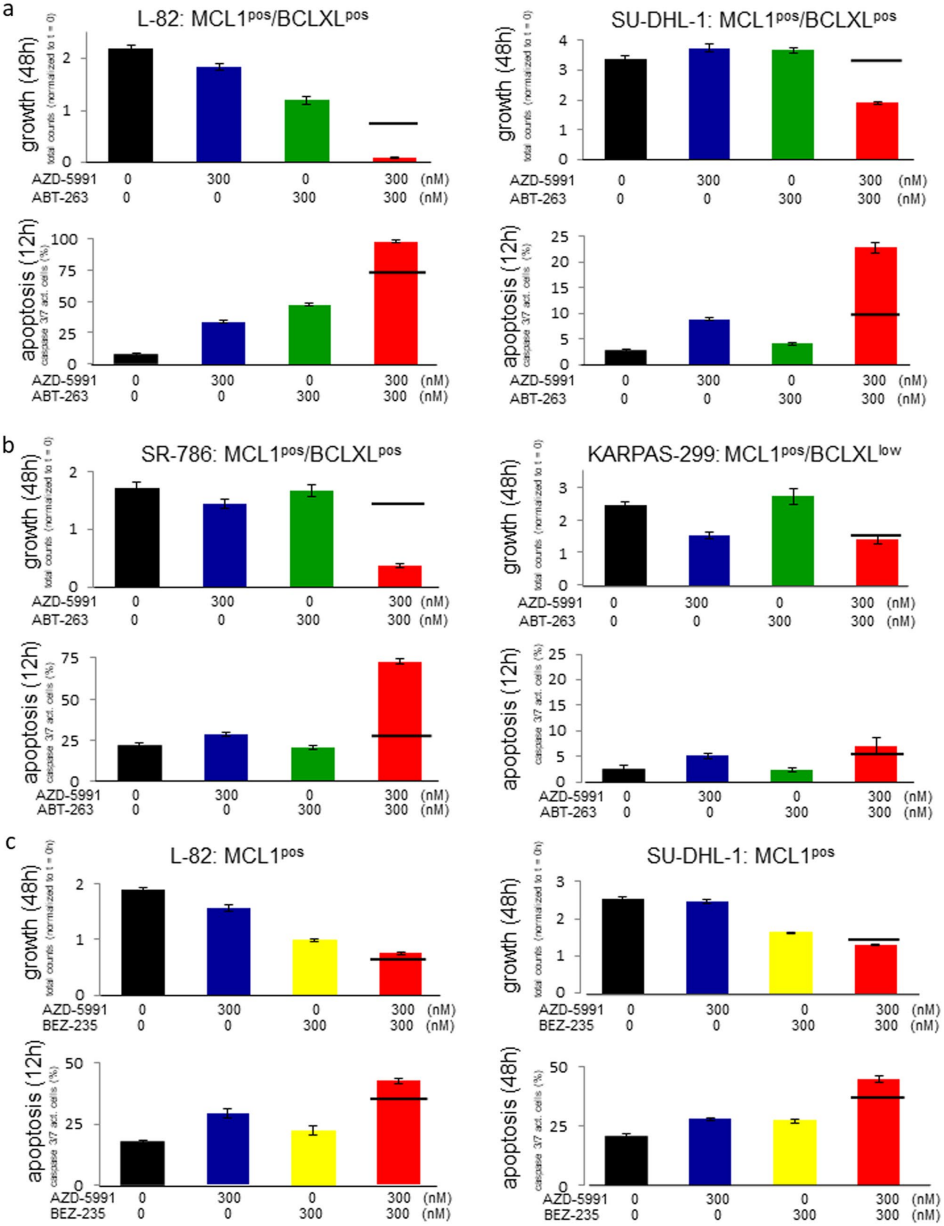
Effect of AZD-5991 and ABT-263 on growth and apoptosis of ALCL cell lines. Life cell imaging data showing the effect of the MCL1 inhibitor AZD-5991 (300 nM) and the BCL2/BCLXL inhibitor ABT-263 (300 nM) on growth (48 h) and apoptosis (12 h) of (**a**) L-82, SU-DHL-1 and (**b**) SR-786, KARPAS-299. (**c**) shows effects of AZD-5991 and BEZ-235 on growth and apoptosis of cell lines L-82 and SU-DHL-1. Apoptosis was assessed by caspase 3/7 activity. The horizontal line shows the expected additive effects of the combination of both inhibitors. Note that the only cell line that does not respond to ABT-263 is the BCLXL^low^ cell line KARPAS-299.

Accordingly, co-stimulation of L-82 cells with suboptimal concentrations of AZD-5991 (300 nM) and ABT-263 (300 nM) yielded synergistic antiproliferative and proapoptotic effects when compared to the effects of each inhibitor alone (Fig. 3a).

Thus, knockdown and inhibitor experiments showed that BCLXL acts as antiapoptotic mediator in cell line L-82, cooperating with MCL1.

At first sight the notion that BCLXL acts as a major antiapoptotic mediator in ALCL seems to conflict with the observation that 5/6 ABT-263 resistant ALCL cell lines (EC50 ≥ 1.25 µM) expressed BCLXL (Fig. 1c, Table 1). These findings provoke the question why BCLXL exhibited an overt antiapoptotic function in L-82, but not in other BCLXL^pos^ cell lines (Fig. 1c, Table 1). Interestingly, also the MCL1^pos^/BCL2^pos^ MM cell line LP-1 tested AZD-5991 sensitive / ABT-263 resistant (Fig. 1c, Table 1), suggesting that MCL1 is the dominant molecule and that inhibitors targeting other antiapoptotic proteins are effective only if MCL1 is also inhibited.

To find out whether indeed ABT-263 resistance of ALCL cell lines resulted from the strong and dominant effect of MCL1, we costimulated all ALCL cell lines with suboptimal concentrations of AZD-5991 and ABT-263.

Regarding growth arrest, the combination of ABT-263 and AZD-5991 achieved more than additive effects in 4/6 BCLXL^pos^ ALCL cell lines (L-82, SR-786, SU-DHL-1, KI-JK) (Fig. 3a,b, Suppl Fig. 4a). Synergistic activation of caspases was observed in even 5/6 (L-82, SR-786, SU-DHL-1, KI-JK, DEL) cell lines (Fig. 3a,b, Suppl. Fig. 4b). Costimulation with both drugs led to growth arrest also in SU-DHL-1 which was found to be resistant to either inhibitor alone (Table 1; Fig. 3a). Cell lines with weak expression levels of BCLXL (ALCL cell line KARPAS-299 and PEL cell line BC-3) did not respond significantly to ABT-263, neither alone nor in combination with the MCL-1 inhibitor (Fig. 3b, Suppl. Fig. 4a,b).

These results provide convincing evidence that besides MCL1 BCLXL acts as second antiapoptotic mediator in ALCL, with dominant effects of MCL1 in most cases.

### BCLXL and BCL2A1: antiapoptotic mediators in ALCL

MCL1 had been described as the main antiapoptotic mediator in ALCL^19^. We show here that BCLXL acts as second antiapoptotic protein in this disease. However, the resistance of cell line SU-DHL-1 to MCL1 and BCLXL inhibitors when applied individually, suggests that still another protective protein might be active in this cell line. qRT-PCR analysis showed that cell line SU-DHL-1 stood out by high levels of BCL2A1 (Fig. 1b). Like BCLXL, BCL2A1 is regulated by the ALCL associated fusion protein NPM-ALK that sustains survival of the malignant cells^24,25^. All ALCL cell lines tested in this study carry the t(2;5)(p23;q35) encoding the NPM-ALK fusion gene.

BCL2A1 knockdown with siRNA oligonucleotides arrested growth and induced apoptosis in SU-DHL-1 (BCL2A1^high^), but not in L-82 (BCL2A1^low^) (Fig. 2b). Supporting the notion that BCL2A1 acts as antiapoptotic mediator in SU-DHL-1, this cell line also responded to the broad BCL2 family inhibitor BI-97C1 (legend to Table 1). BI-97C1 (Sabutoclax) is a drug which among other BCL2 family members also targets BCL2A1^26^.

In conclusion, knockdown and inhibitor experiments show that MCL1 is not the only antiapoptotic BCL2 family member in ALCL. BCLXL and BCL2A1 protect from apoptosis as well. If validated in primary cells, our data suggest that treatment with a combination of inhibitors against MCL1, BCLXL and BCL2A1 might be an alternative option for patients.

### Inhibition of PI3K/mTOR

The combination of specific BH3 mimetics or the use of inhibitors targeting multiple BCL2 family members might be a strategy for the treatment of ALCL. However, targeted therapies with BCLXL inhibitors have been associated with thrombocytopenia^27^. Therefore, we set out to elucidate whether the combination of the MCL1 inhibitor AZD-5991 and BEZ-235, the latter a dual inhibitor of PI3K and mTOR, might be an alternative to induce apoptosis in cells that are resistant to the MCL1 inhibitor alone.

The rationale for these experiments lies in earlier studies reporting that apoptosis in ALCL and DLBCL models was induced by downregulation of MCL1 following inhibition of the AKT/mTOR pathway^28,29^. Thus, we applied a dual PI3K/mTOR inhibitor (BEZ-235) to test whether this drug would repress MCL1 in our cell lines. We speculated that a double hit against MCL1, reducing expression (BEZ-235) and inhibiting its function (AZD-5991), might create cooperative effects.

**Figure 4:**
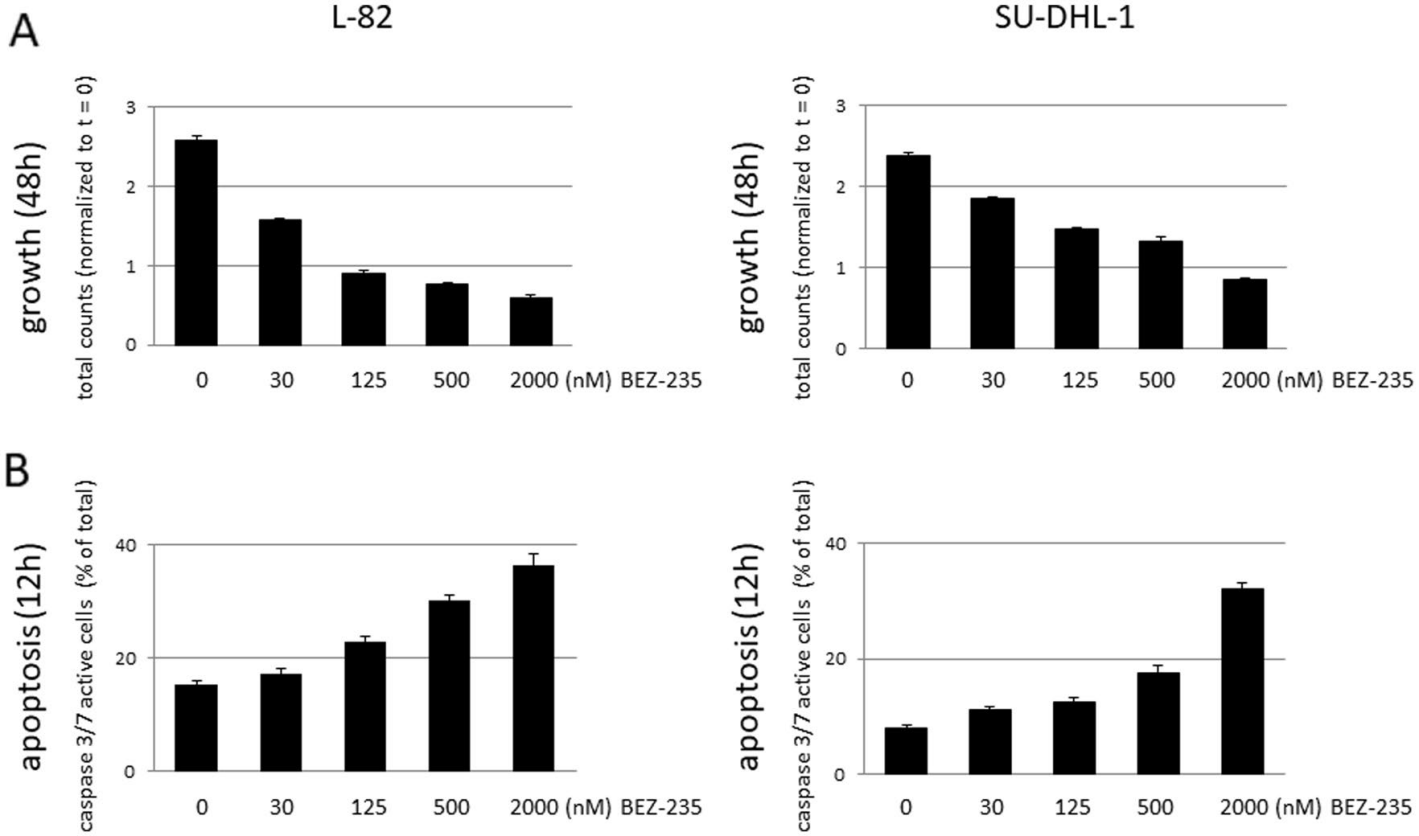
Effect of BEZ-235 on growth and apoptosis of ALCL cell lines. Life cell imaging data showing the effect of the PI3K/mTOR inhibitor BEZ-235 on (**a**) growth (48 h) and (**b**) apoptosis (12 h) of ALCL cell lines L-82 and SU-DHL-1. Apoptosis was assessed by caspase 3/7 activity.

**Figure 5:**
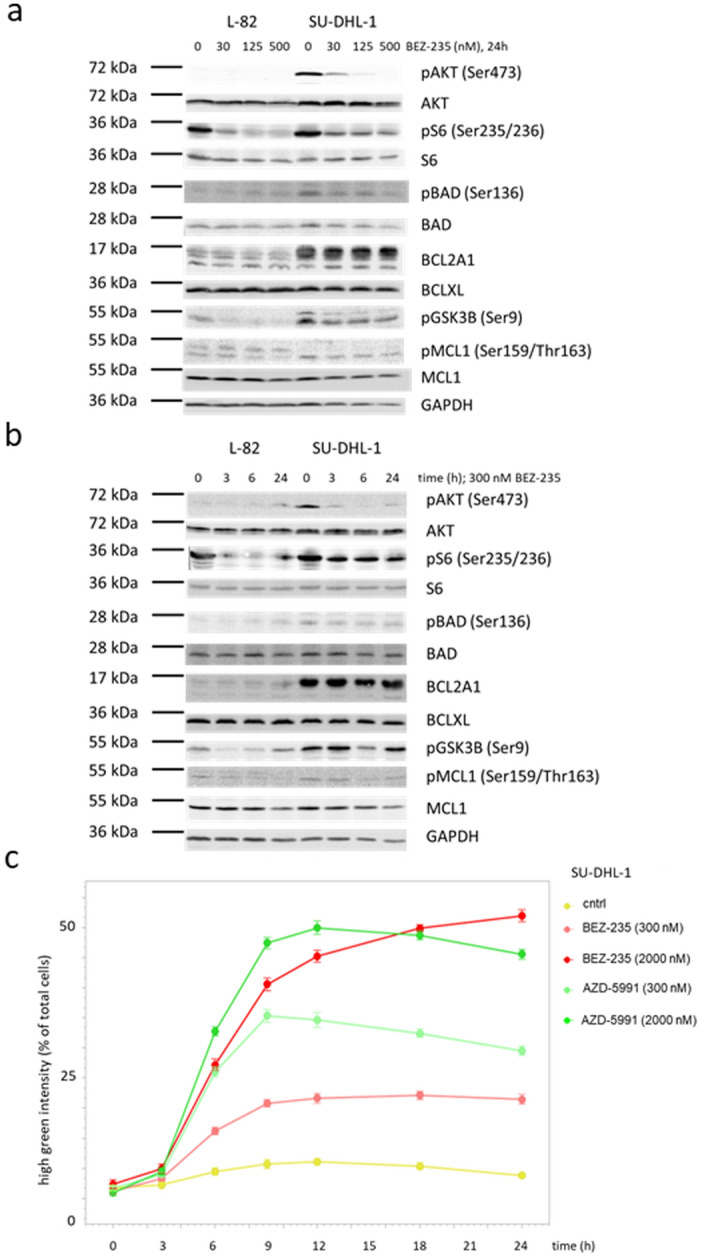
Effect of BEZ-235 on PI3K/AKT activity and on expression of BCL2 family members. Western blots (12% PAA gel) showing (**a**) dose- and (**b**) time-dependent effects of BEZ-235 on the expression of BCL2 family members and on phosphorylation of AKT, the mTOR target S6 and the BCL2 family member BAD. Dose-dependent effects were assessed after 24 h, time-dependent effects with a concentration of 300 nM BEZ-235. (**c**) Kinetics of apoptosis induced by BEZ-235 and AZD-5991 as determined by caspase 3/7 activity. Note that the kinetics of caspase 3/7 activity stimulated by BEZ-235 and AZD-5991 do not differ.

Indeed, BEZ-235 inhibited proliferation (48 h) and induced apoptosis (12 h) in a dose-dependent manner (Fig. 4). Low concentrations of BEZ-235 (30 nM, 24 h) induced dephosphorylation of AKT, the AKT target GSK3B and of the mTOR target S6 (Fig. 5a). Induction of apoptosis as assessed by caspase 3/7 activation paralleled dephosphorylation of AKT and its targets (Fig. 5b,c). However, apoptosis was not preceded by downregulation of MCL1 which we had expected based on the previously reported data^28,29^ (Fig. 5a,b). The MCL1 protein has a very short half-life^30,31^. Consequently, stimulation of cells with the global transcriptional inhibitor THZ1 (200 nM, 7.5 h) resulted in a dramatic drop of MCL1 (Suppl. Fig. 5). Even at high concentrations (500 nM, 24 h), BEZ-235 did not have such an effect on the expression of MCL1 (Fig. 5a). Also BCLXL and BCL2A1 were not influenced by BEZ-235 (Fig. 5a,b). Therefore, we conclude that the BEZ-235 induced apoptosis in ALCL cell lines was not caused by downregulation of MCL1, and also not by downregulation of BCLXL and BCL2A1.

BEZ-235 (300 nM, 6 h) induced expression of several dozen genes in cell lines L-82 and SU-DHL-1 (Suppl. Table 2A,B). However, the upregulated genes are not downstream targets of FOXO1 (FKHR) and FOXO3 (FKHRL1), transcription factors which are inactivated by AKT-mediated phosphorylation^32,33^. Especially, we could not observe upregulation of the proapoptotic FOXO target BIM (Suppl. Fig. 6).

BAD is another target of AKT. Dephosphorylated BAD binds to the antiapoptotic BCL2 and BCLXL molecules, replacing proapoptotic BAX which then triggers apoptosis^34–37^. However, effects of BEZ-235 on phosphorylation of BAD could not be observed (Fig. 5a,b).

Thus, we could show that BEZ-235 induced dephosphorylation of AKT and of the mTOR target S6 in ALCL cell lines, but the downstream effectors mediating apoptosis still remain elusive. Important in the context of this study is that BEZ-235 and AZD-5991 showed additive effects on ALCL cell lines regarding arrest of cell growth and induction of apoptosis (Fig. 3c).

In sum, our data suggest that MCL1 inhibitors might be useful for the treatment of PEL. Furthermore, the combination of PI3K/AKT/mTOR pathway inhibitors and of MCL1 inhibitors might be an alternative for treatment of patients with ALCL.

## Methods

### Cell lines

Cell lines were taken from the stock of the cell lines bank (Leibniz Institute DSMZ – German Collection of Microorganisms and Cell Cultures). Cell lines were authenticated by DNA profiling and cytogenetics. Detailed references and cultivation protocols have been described previously^38^.

### RNA-sequencing analysis

Total RNA was extracted via miRNeasy Mini Kit (Qiagen, Hilden, Germany) including DNase digestion. Library preparation and sequencing were commissioned to GATC Biotech (Cologne, Germany). RNA expression analysis was performed as described previously^6^. RNA-seq data were deposited at ArrayExpress E-MTAB-7721. For the heatmap visualization 0.1 was added to normalized expression levels prior to log2 transformation.

### Growth and apoptosis

About 2 × 10^4^ cells (in 50 µl) were seeded in triplicate in 96-well flat bottom microtiter plates. Effectors were added as 2 × concentrated solutions in a 50 µl volume. Growth and apoptosis were determined with the IncuCyte S3 Live-Cell analysis System (Sartorius, Essen Bioscience, Welwyn Garden City, UK). We used the Caspase 3/7 reagent (Essen Bioscience #4704) to detect apoptotic cells.

### Knock-down experiments

Transfection with siRNA oligonucleotides was performed as described previously^39^. Gene-specific siRNA oligonucleotides and AllStars negative control siRNA were obtained from Qiagen. The siRNA (80 pmol) was transfected into 1 × 10^6^ cells by electroporation using an EPI-2500 impulse generator (Fischer, Heidelberg, Germany) at 350 V for 10 ms. Treated cells were harvested after 24 h or 48 h.

### Quantitative real-time PCR analysis

RNA was prepared using the RNeasy Mini kit (Qiagen). For mRNA quantification, reverse transcription was performed using the SuperScript II reverse transcriptase kit (Invitrogen, Karlsruhe, Germany). PCR was performed on a 7500 Applied Biosystems (Darmstadt, Germany). TaqMan probes (Applied Biosystems) were used to quantify human *BAX* (Hs00180269_m1), *BCL2* (Hs00153350_m1), BCL2A1 (Hs00187845_m1), *BCLW* (Hs00187848_m1), *BCLXL* (Hs00236329_m1), CDKN1A (Hs00355783_m1), *MCL1* (Hs03043899_m1), *MDM2* (Hs01066930_m1) and *NOXA1* (Hs00611456_g1) using *TBP* as endogenous control. DNA was isolated with the High Pure PCR Template Preparation Kit (Roche Diagnostics, Mannheim, Germany). Quantitative genomic PCR was performed with SYBR-green reagents (Applied Biosystems). Primers and PCR conditions for genomic PCR are shown in Table S3. Relative expression levels were calculated using the ΔΔCt method.

### Western blot analysis

Samples were prepared as described previously^40^. Anti AKT (#4691), pAKT (Ser473) (#4060), BAD (#9239), pBAD (Ser136) (#4366), BCL2 (#4223), BCL2A1 (#14093), BCL-W (#2724), BCLXL (#2764), pGSK3B (Ser9) (#9323), MCL1 (#39224), pMCL1 (Ser159/Thr163) (#4579), S6 (#2317) and pS6 (Ser235/236) (#2211) antibodies were purchased from Cell Signaling (New England Biolabs, Frankfurt, Germany). Anti GAPDH (ab8245) antibody was from Abcam (Cambridge, UK). Specific bands on nitrocellulose membranes were visualized with the biotin/streptavidin–horseradish peroxidase system (GE Healthcare, Little Chalfont, UK) in combination with the “Renaissance Western Blot Chemoluminescence Reagent” protocol (Perkin Elmer, Waltham, MA, USA).

### Expression array

Cell lines L-82 and SU-DHL-1 were stimulated with BEZ-235 (300 nM; 0 h, 3 h, 6 h). RNA was isolated using the RNeasy Mini kit (Qiagen). Quality and integrity of total RNA was controlled on Agilent Technologies 2100 Bioanalyzer (Agilent Technologies; Waldbronn, Germany). For biotin labelling 2–10 ng of total RNA were used according to GeneChip Pico Kit (Affymetrix). Then, 5.5 µg of biotinylated cDNA were fragmented and placed in a hybridization cocktail containing four biotinylated hybridization controls (BioB, BioC, BioD, and Cre) as recommended by the manufacturer. Samples were hybridized to an identical lot of Affymetrix Clariom S (400 Format) for 17 h at 45 °C. Hybridization was done for 16 h at conditions recommended by the manufacturer. Clariom S chips were washed and stained in the Affymetrix Fluidics Station 450. GeneChips were scanned using the Affymetrix GCS 3000. Image analysis was done by Affymetrix GeneChip Command Console Software (AGCC) and Affymetrix Expression Console Software. Clariom S arrays were analyzed via R/Bioconducor using the package oligo including multi-array average (RMA) background correction, quantile normalization and log2 transformation, whereas the annotation file was retrieved from Thermofisher (version na36, hg38, transcripts). Genes were filtered to at least log2FC < -1 in control versus 6 h after BEZ-235 treatment. The data were deposited with ArrayExpress under E-TAB-10903.

## Supplementary Information

Supplementary Information.
