# Supplementary material for: Inhibition of MCL1 induces apoptosis in anaplastic large cell lymphoma and in primary effusion lymphoma
Source: Sci Rep. 2022 Jan 20;12:1085. doi: 10.1038/s41598-022-04916-6 (PMC8776734; doi:10.1038/s41598-022-04916-6)
Supplement: Supplementary file 1 — Supplementary Information. [file 41598_2022_4916_MOESM1_ESM.pdf]

Suppl. Figure 1a

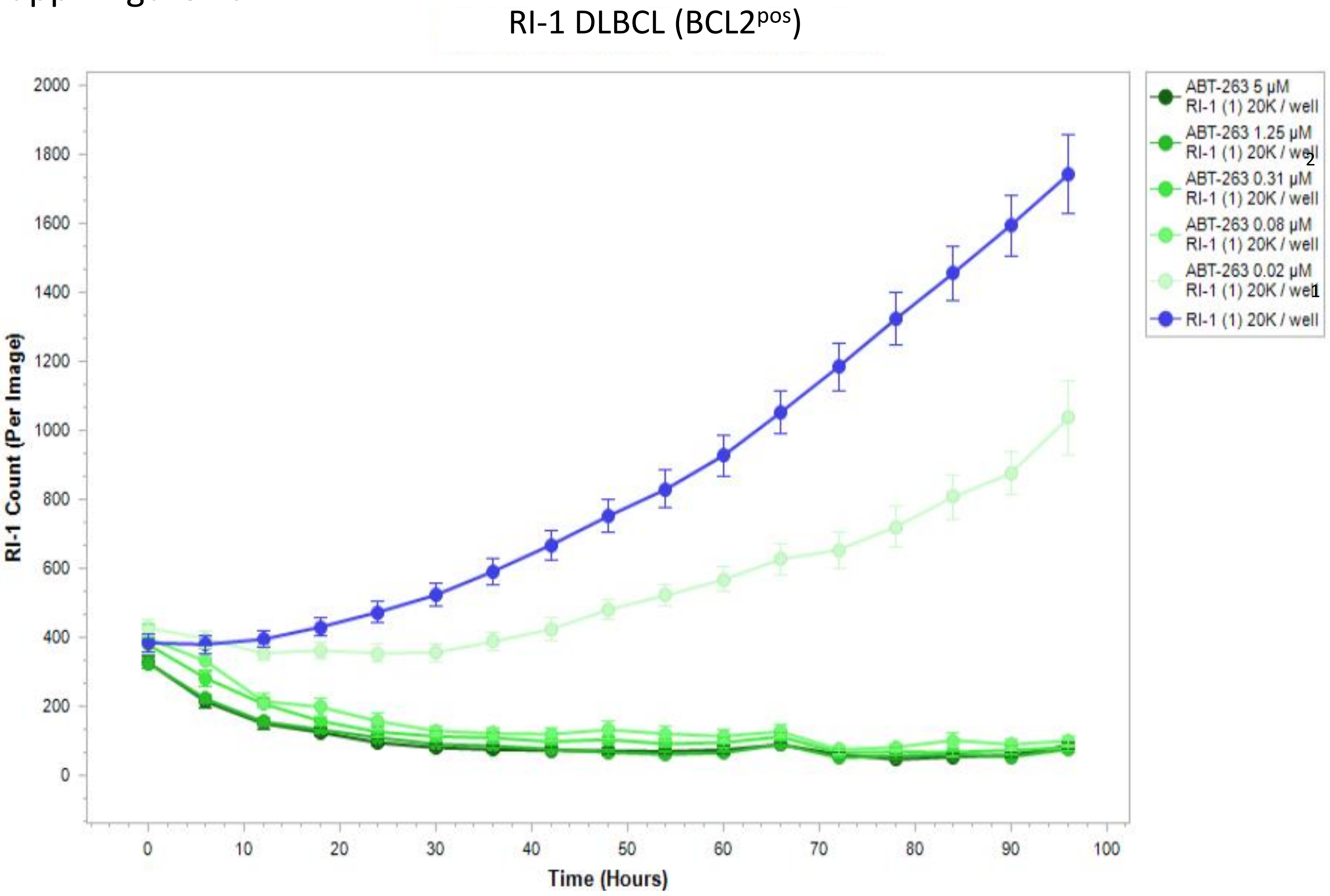

Suppl. Figure 1b

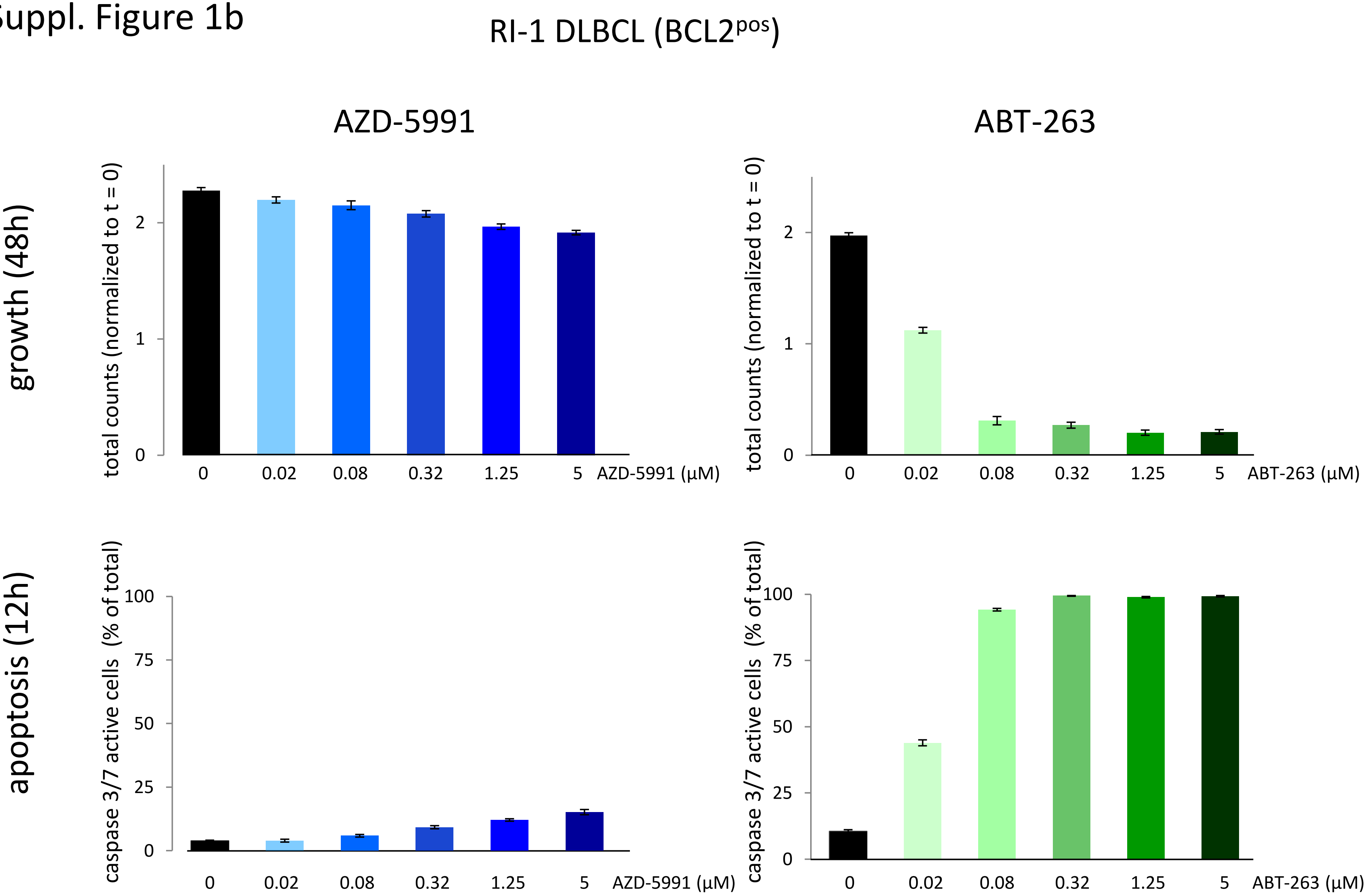

Suppl. Figure 1c

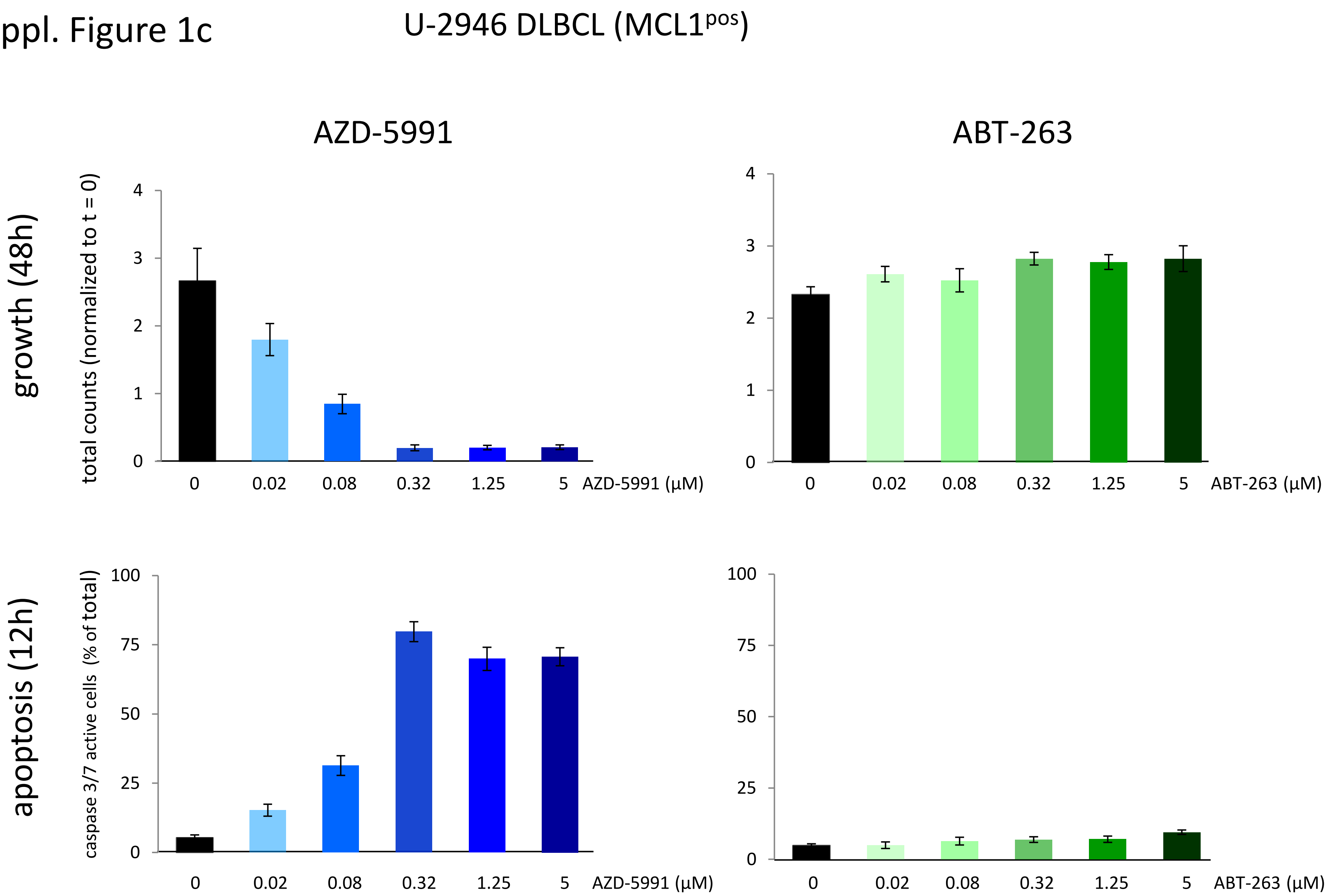

Suppl. Figure 2

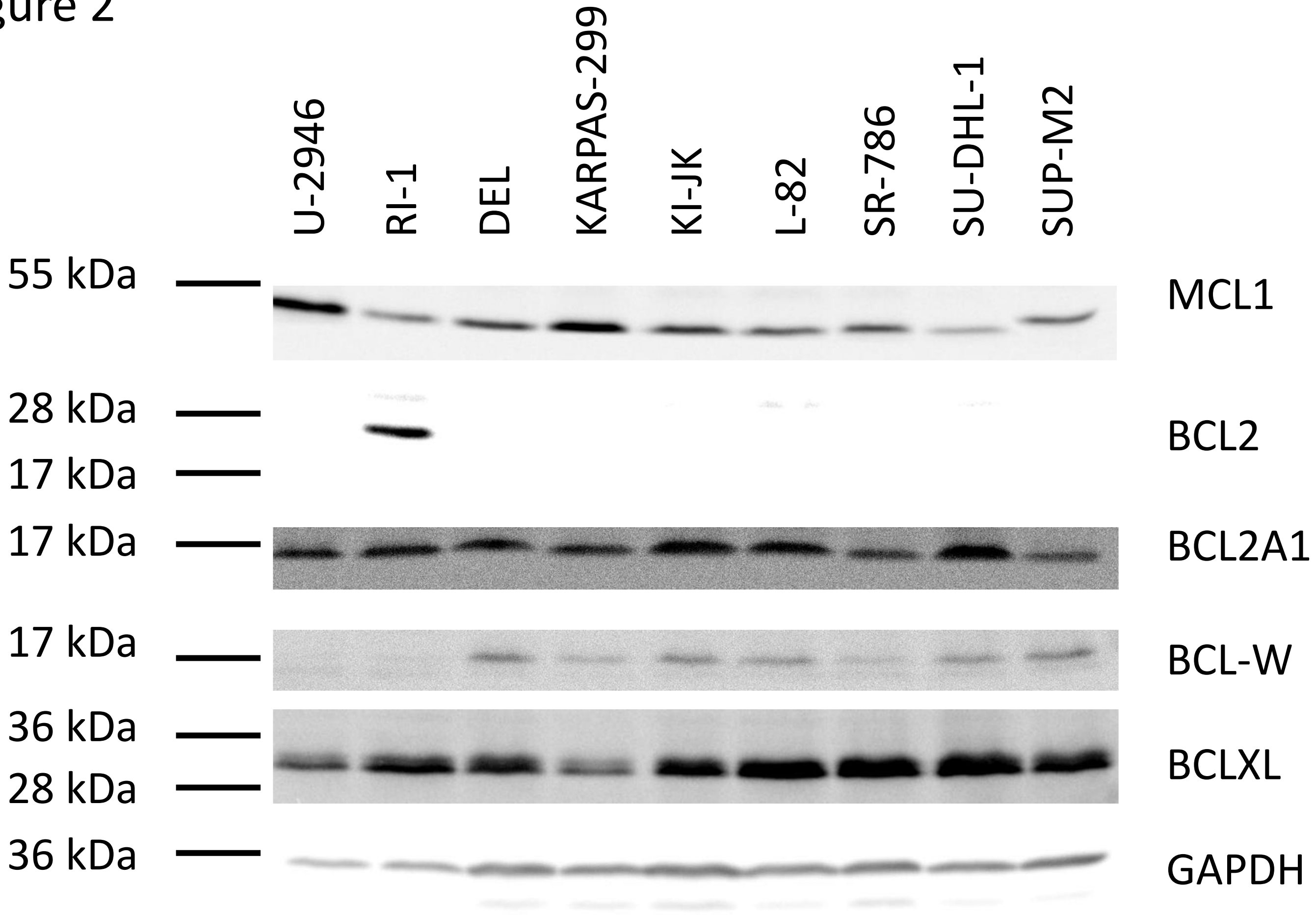

Suppl. Figure 3

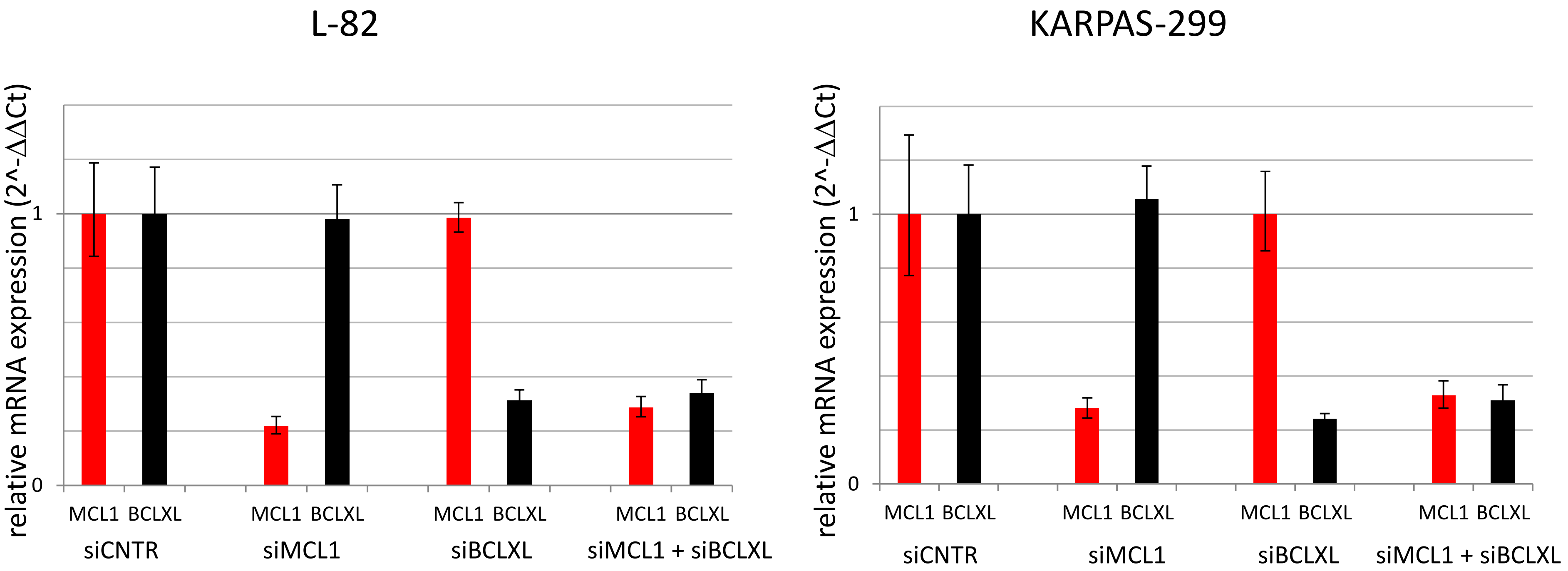

Suppl. Figure 4a

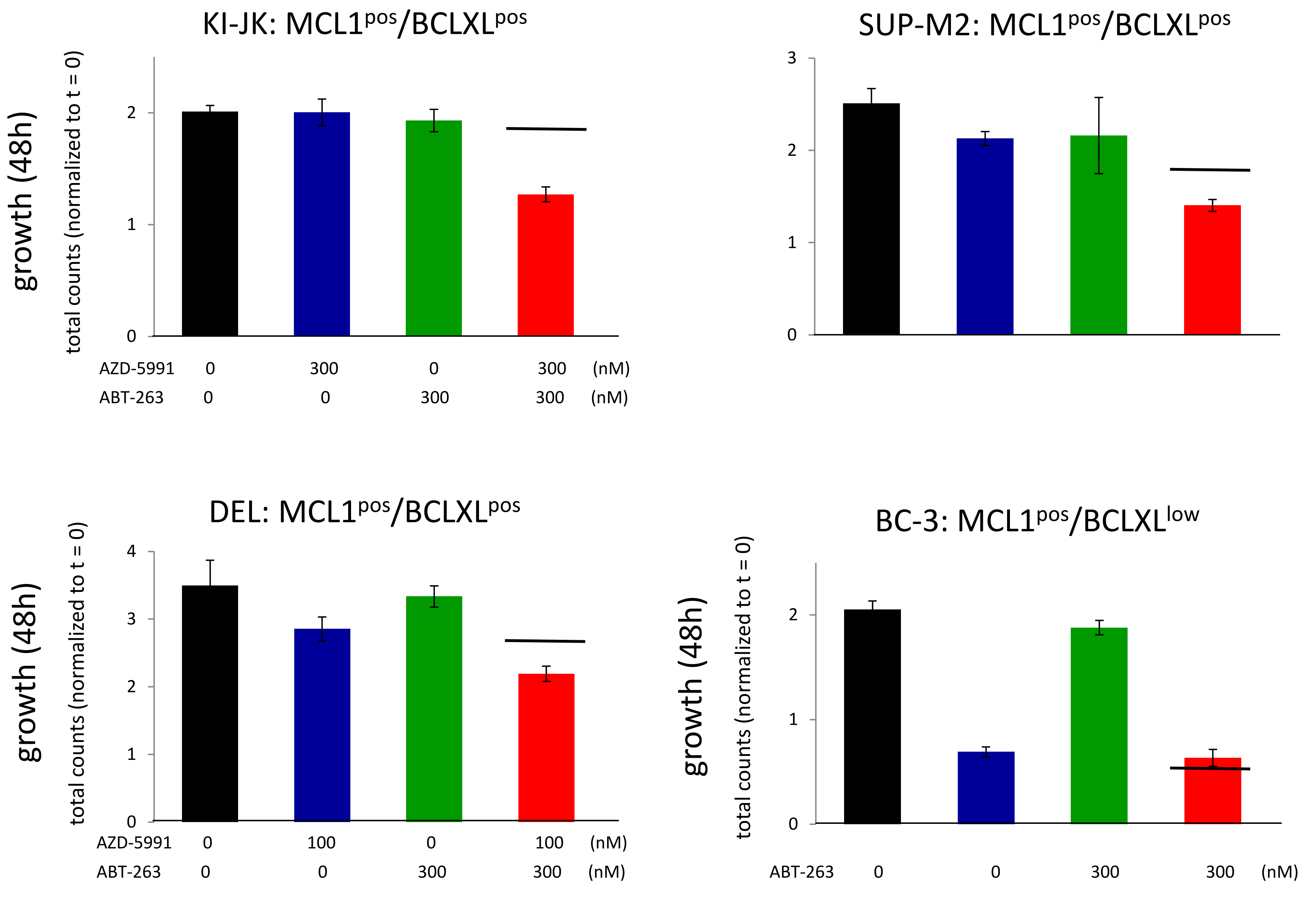

Suppl. Figure 4b

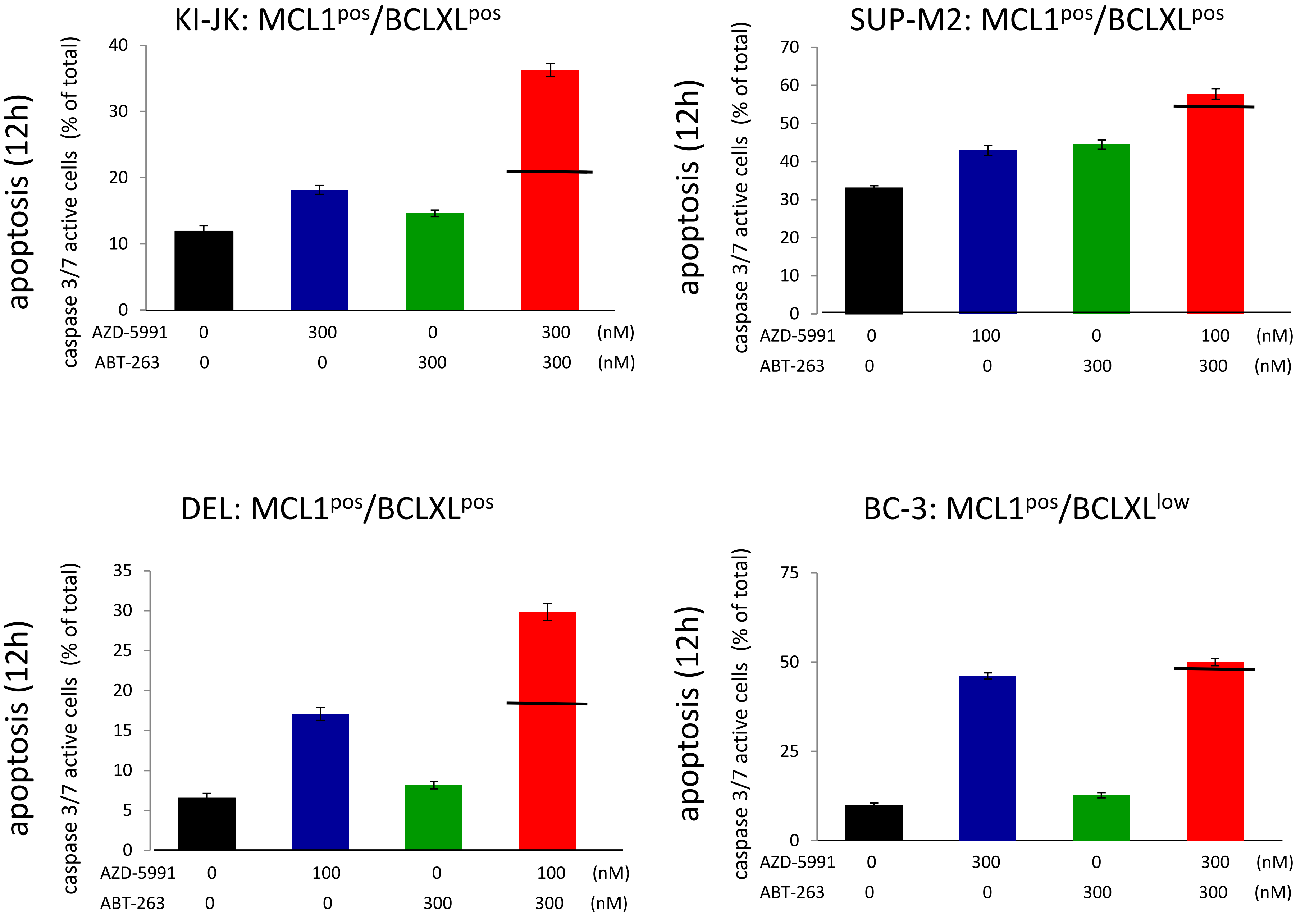

Suppl. Figure 5

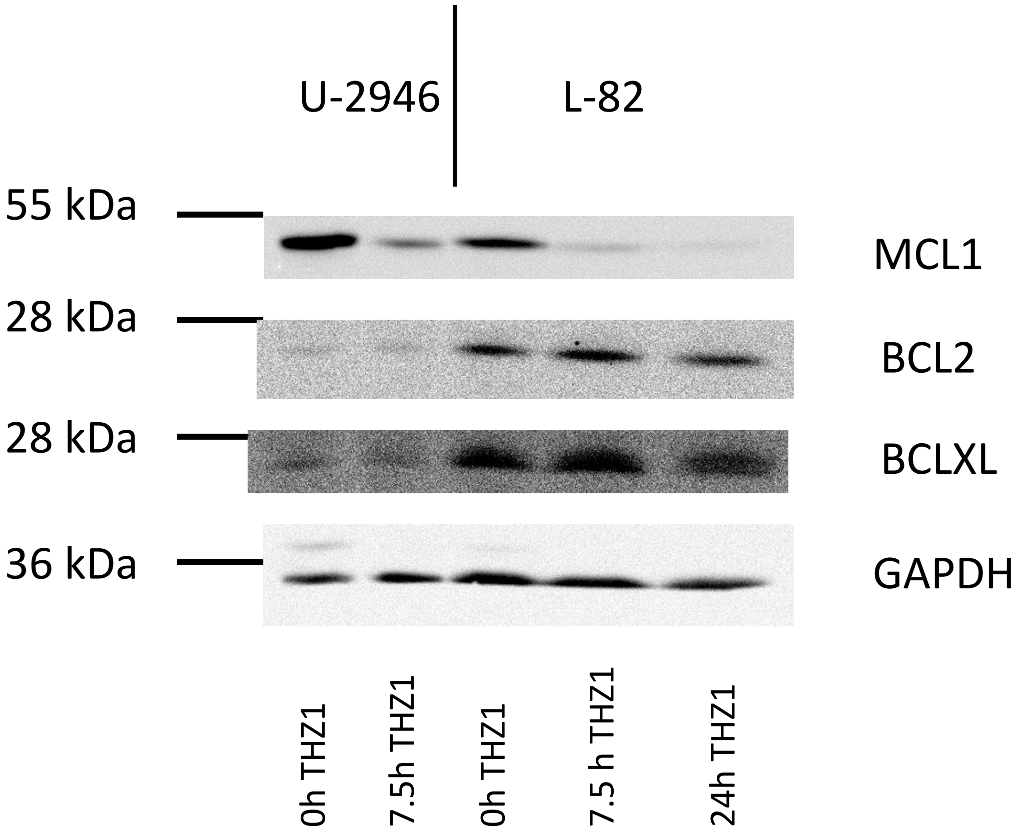

Suppl. Figure 6

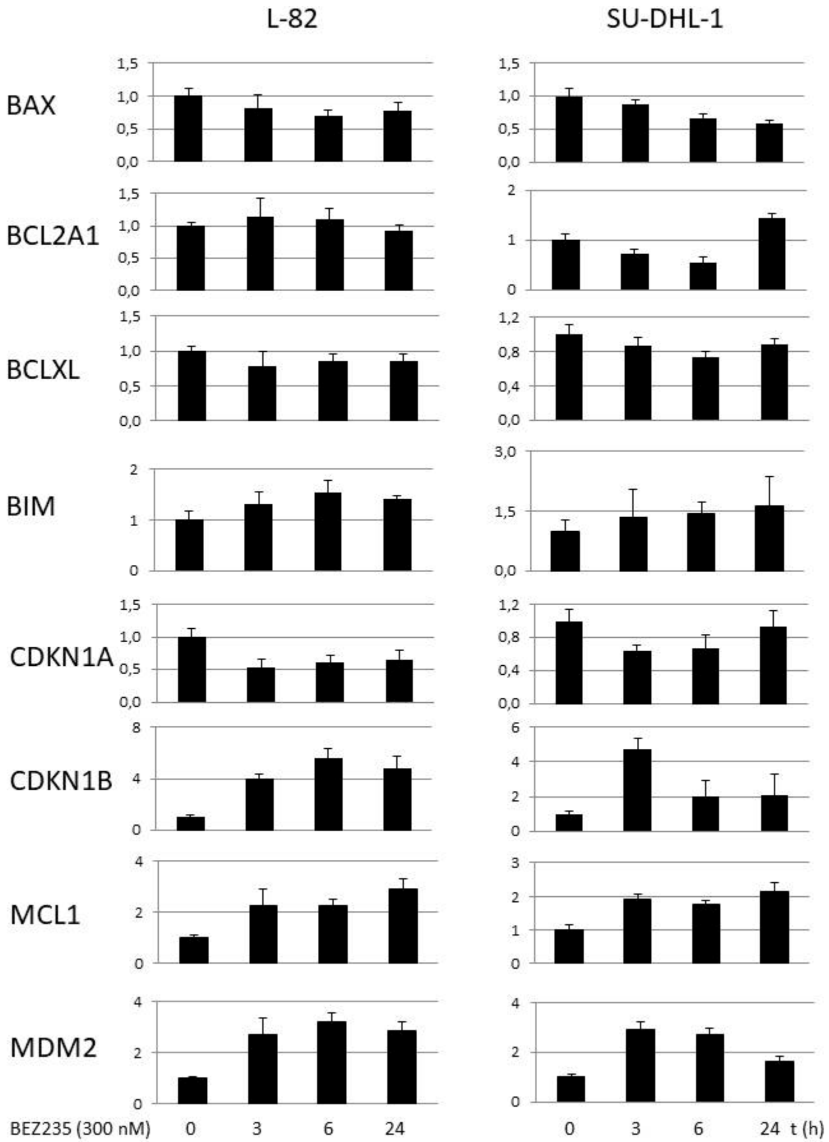

Suppl. Figure 7

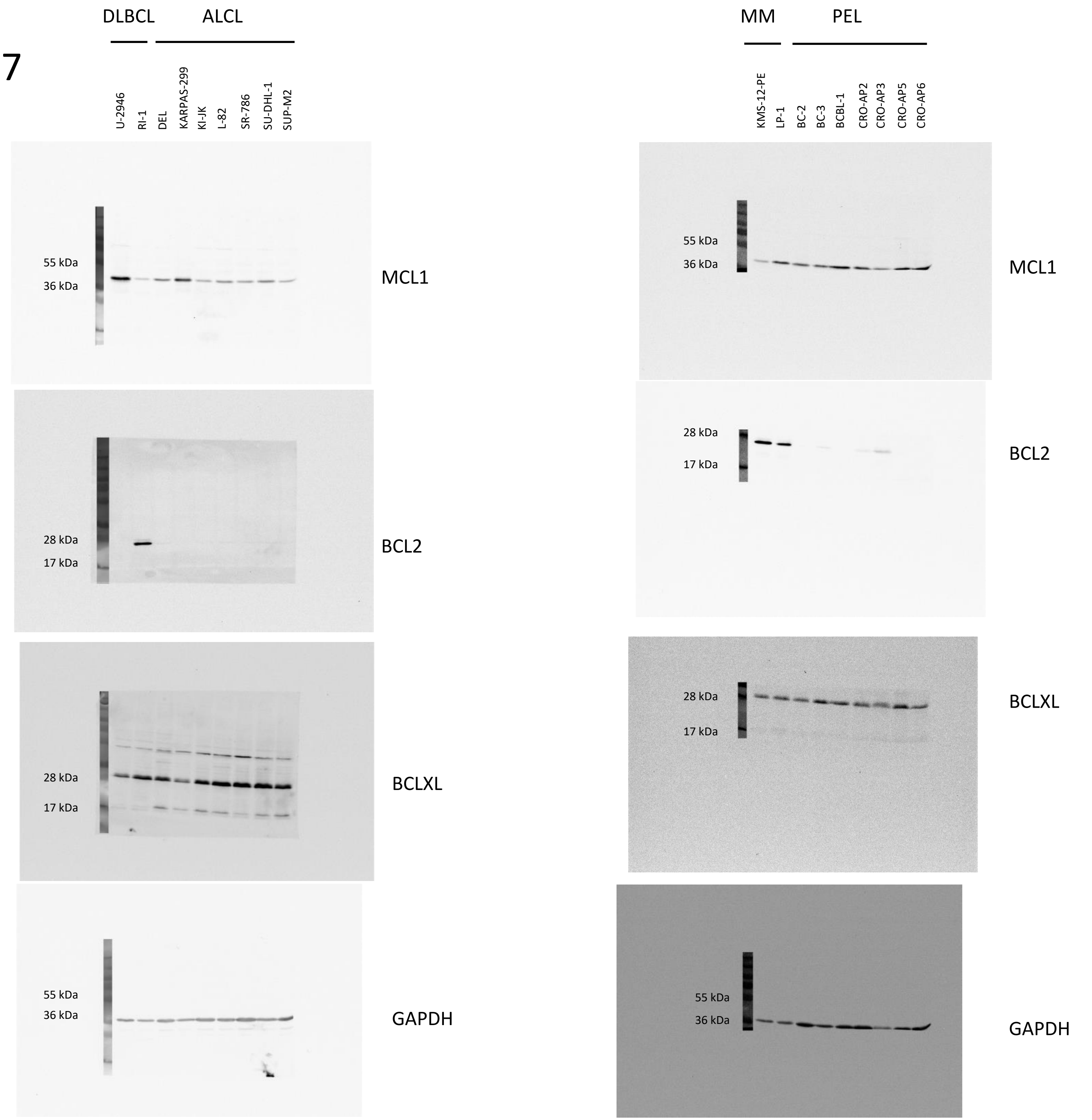

Suppl. Figure 8

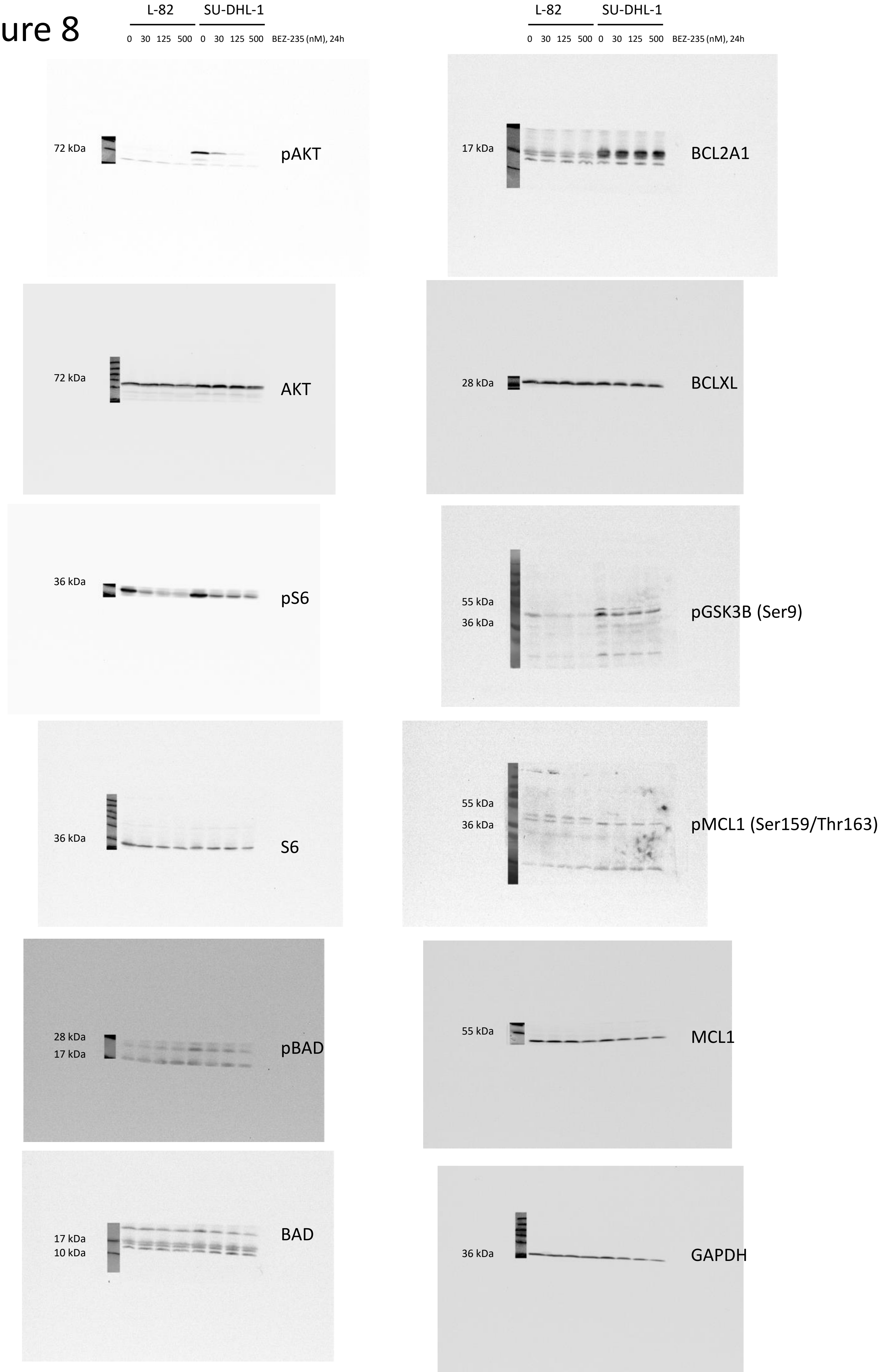

Suppl. Figure 9

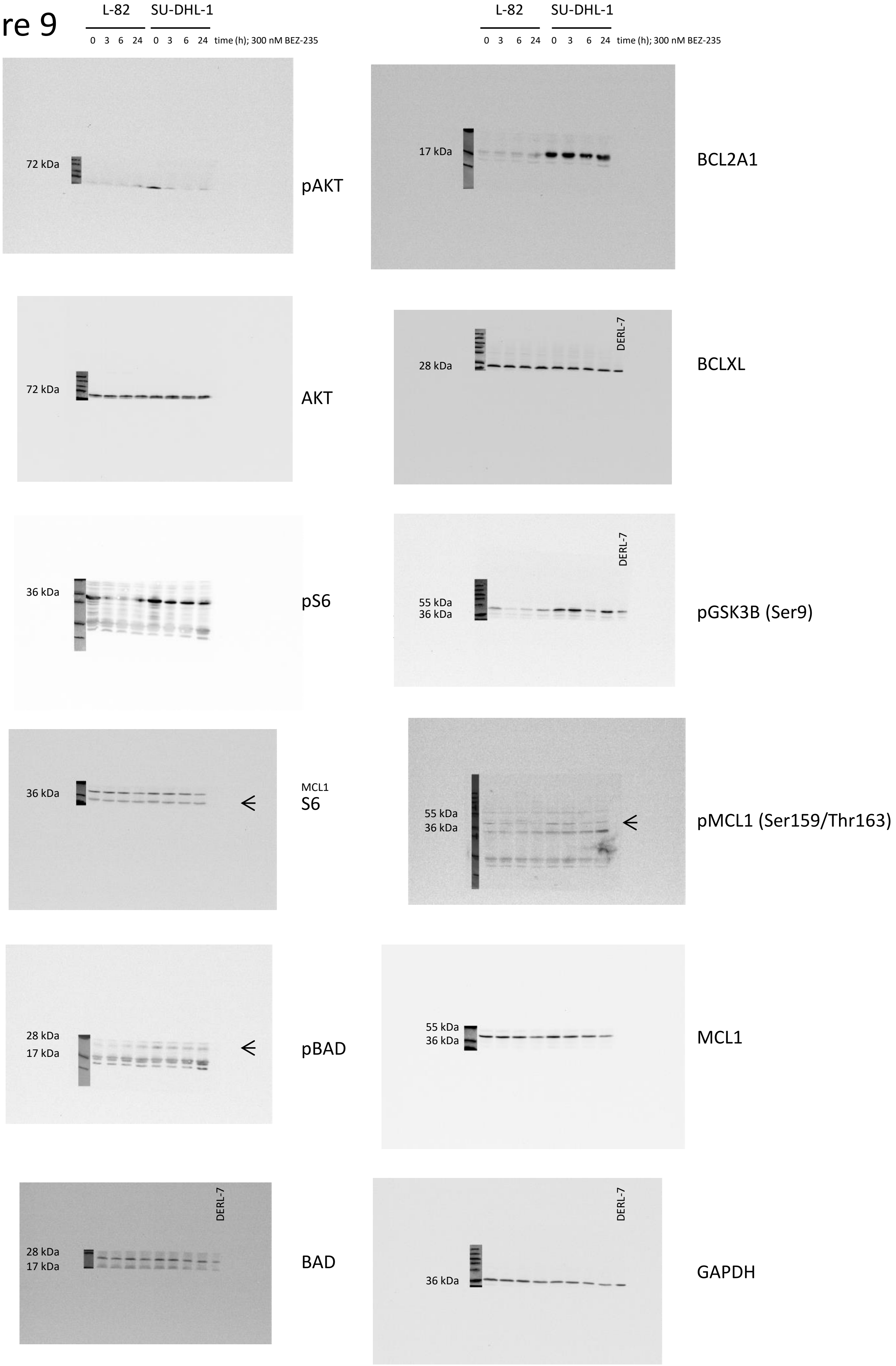

Suppl. Figure 10

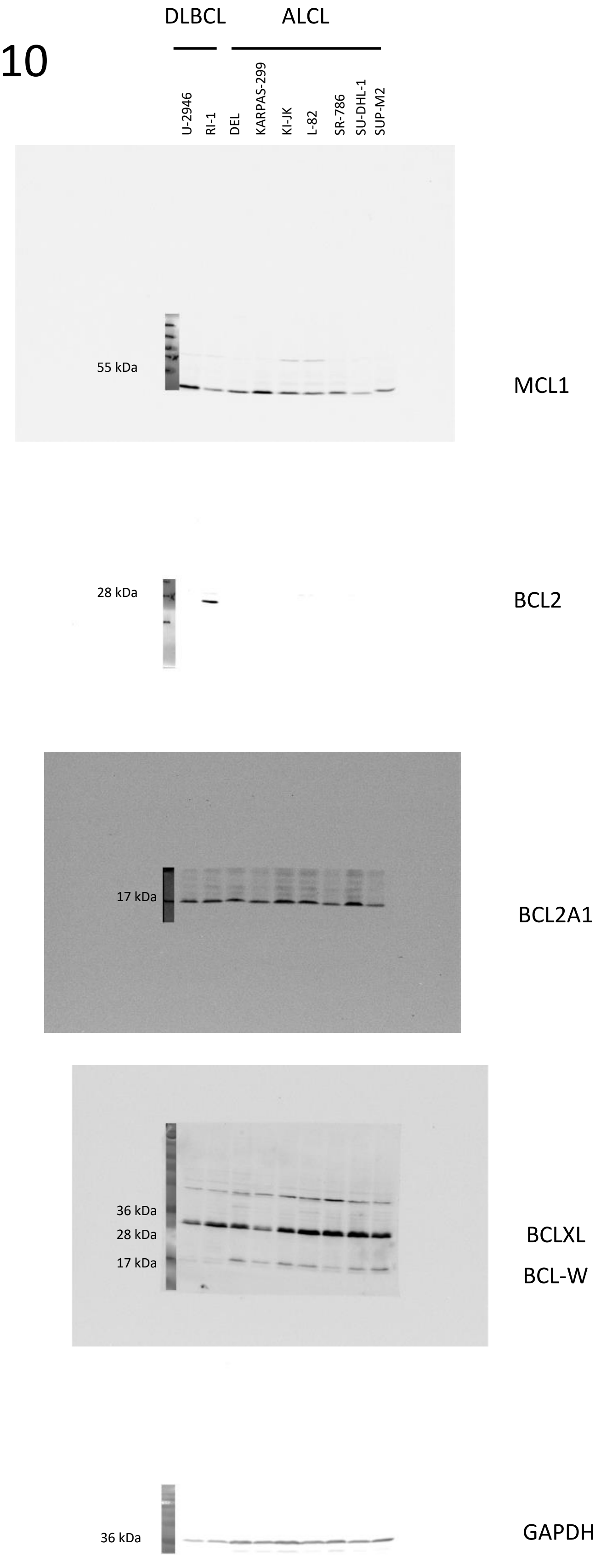

Suppl. Figure 11

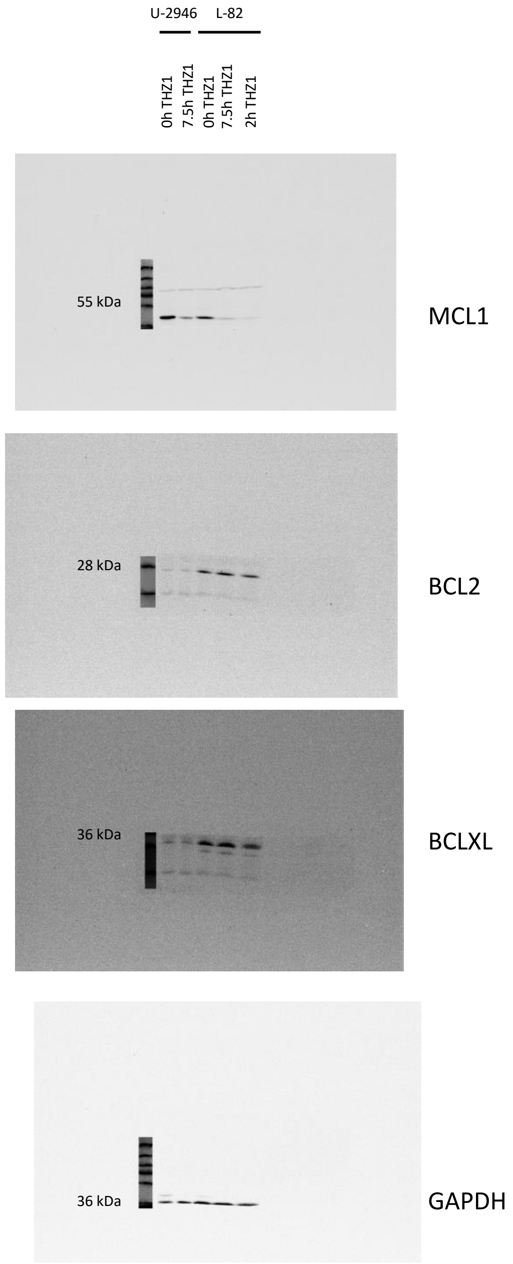

Supplementary Files

Supplementary Figure 1. Effect of AZD-5991 and ABT-263 on growth and apoptosis of DLBCL cell lines. a) Life cell imaging data showing kinetics of the dose-dependent effects of ABT-263 on cell numbers of the BCL2^{pos} DLBCL cell line RI-1. Antiproliferative and proapoptotic effects of the MCL-1 inhibitor AZD-5991 and of the BCL2 inhibitor ABT-263 on the BCL2^{pos} cell line RI-1 (b) and on the MCL1^{pos} cell line U-2946 (c). Apoptosis was assessed by caspase 3/7 activity.

Supplementary Figure 2. Expression of BCL2 family members in ALCL. Western blot analysis showed the expression pattern of BLC2 family members in ALCL cell lines.

Supplementary Figure 3. Efficiency of MCL1 and BCLXL knockdown. qRT-PCR confirmed the efficiency of MCL1 and BCLXL knockdown (24h) with siRNA oligonucleotides.

Supplementary Figure 4. Effect of AZD-5991 and ABT-263 on growth and apoptosis of ALCL cell lines. Life cell imaging data showing the effect of the MCL1 inhibitor AZD-5991 (300 nM) and the BCL2/BCLXL inhibitor ABT-263 (300 nM) on a) growth (48h) and b) apoptosis (12h) of ALCL cell lines. Apoptosis was assessed by caspase 3/7 activity. The horizontal line shows the expected additive effects of the combination of both inhibitors. Note that the only cell line that does not respond to ABT-263 is the BCLX^{low} PEL cell line BC-3.

Supplementary Figure 5. Effect of transcriptional inhibition on MCL1 expression. Western blot analysis showed that stimulation with the transcriptional

inhibitor THZ1 (200 nM) induced a rapid decrease of MCL1 levels in the DLBCL cell line U-2946 and in the ALCL cell line L-82.

Supplementary Figure 6. Effect of BEZ-235 on mRNA expression levels of BCL2 family members and of TP53 targets. qRT-PCR analysis showing time-dependent effects of BEZ-235 (300 nM) on mRNA expression of BCL2 family members and on potential down-stream targets of AKT. Note that the FOXO target BIM is not upregulated after stimulation with BEZ-235. Also TP53 targets CDKN1A and NOXA (NOXA with ct > 40) are not induced by BEZ-235.

Supplementary Figure 7. Original blots cropped and presented in Figure 1C. Note that in some cases blots were cut to allow the simultaneous use of two antibodies.

Supplementary Figure 8. Original blots cropped and presented in Figure 5A. Blots were cut to allow the simultaneous use of several antibodies.

Supplementary Figure 9. Original blots cropped and presented in Figure 5B. Blots were cut to allow the simultaneous use of several antibodies. On several blots, lysate of cell line DERL-7 was included as positive control.

Supplementary Figure 10. Original blots cropped and presented in Supplementary Figure 2. Note that blots were cut to allow the simultaneous use of several antibodies.

Supplementary Figure 11. Original blots cropped and presented in Supplementary Figure 5. Note that blots were cut to allow the simultaneous use of several antibodies.

Supplementary Table 1. Amplification status of *MCL1* (red), *BCL2* (black) and *BCLXL* (blue)

Disease / cell line	karyotype	CGH array	q gen PCR
**ALCL**			NC-NC_LINE1
DEL	(2n)	2n / 1n / 2n	2n / 1n / 2n
KARPAS-299	(2n)	n.d. / n.d.	9n / 2n / 2n
KI-JK	(3n) +1,+1, +20	n.d. / n.d.	6n / 2n / 4n
L-82	(3n), +1	2.5n / 1.5n / 4n	3n / 1.5n / 3.5n
SR-786	(3n), +1,-18	2.5n / 1n / 4n	3n / 1n / 4n
SU-DHL-1	(3n) +1,-18, -20	2.5n / 1n / 4n	3n / 1.5n / 2n
SUP-M2	(2n), +1	2.5n / 2n / 2n	2.5n / 2n / 2n
**DLBCL**			
RI-1	(2n)	n.d. / n.d.	2n / 18n / 2n
U-2946	(2n)	6n / 2n / 2n	14n / 2n / 2n
**MM/PCL**			
ARH-77	(2n)	n.d.	3n / 2n / 2n
KMS-12-PE	(2n)	n.d.	3n / 9n / 2n
L-363	(2n)	n.d.	3n / 2n / 2n
LP-1	(3n) -1, +18, +20	n.d.	8n / 3n / 4n
MOLP-8	(2n)	n.d.	3n / 2n / 2n
OPM-2	(3n) +1, +1, +1, +18	n.d.	5n / 2n / 2n
U-266	(2n)	n.d.	3n / 2n / 2n
**PEL**			
BC-1	(2n)	n.d.	3n / 1n / 2n
BC-2	(4n) -18	n.d.	2n / 1n / 2n
BC-3	(2n)	n.d.	2n / 1.5n / 2n
BCBL-1	(4n)	n.d.	3n / 1n / 2n
CRO-AP2	(2n)	n.d.	3n / 1.5n / 2n
CRO-AP3	(4n) -1	2.5n / 2n / 2n	3n / 1.5n / 2n
CRO-AP5	(2n)	n.d.	3n / 1.5n / 2n
CRO-AP6	(2n)	n.d.	3n / 1.5n / 2n

MCL1 (chr1q21.3), BCL2 (chr18q21.33), BCLXL (chr20q11.21); for detailed karyotypes see www.dsmz.de;

q gen PCR: endogenous control: LINE1; diploid reference cell line: NC-NC; n.d. not done.

Supplementary Table 2A: Genes upregulated in SU-DHL-1 after stimulation with BEZ-235 (300 nM)

transcript_cluster_id	seqname	symbol	description	gb	SUDHL1_c	SUDHL1_BEZ_3h	SUDHL1_BEZ_6h	log2FC_SU_c_vs_3h	log2FC_SU_c_vs_6h
TC1100012131.hg.1	chr11	MMP1	matrix metallopeptidase 1	NM_001145938	6.69	11.58	11.54	-4.89	-4.85
TC0400007771.hg.1	chr4	HTN3	histatin 3	NM_000200	4.28	7.55	7.31	-3.27	-3.03
TC0100007127.hg.1	chr1	ACTL8	actin-like 8	NM_030812	5.23	8.68	8.21	-3.45	-2.98
TC0500009301.hg.1	chr5	GABRA6	gamma-aminobutyric acid (GABA) A receptor, alpha 6	NM_000811	4.09	7.24	7.03	-3.14	-2.93
TC1900008544.hg.1	chr19	RCN3	reticulocalbin 3, EF-hand calcium binding domain	NM_020650	6.00	9.10	8.89	-3.10	-2.90
TC0100012149.hg.1	chr1	FMN2	formin 2	NM_001305424	4.68	7.41	7.56	-2.73	-2.89
TC0200008260.hg.1	chr2	VAMP8	vesicle associated membrane protein 8	NM_003761	6.27	9.10	8.94	-2.83	-2.67
TC1200007359.hg.1	chr12	CNTN1	contactin 1	NM_001256063	5.97	8.66	8.54	-2.69	-2.56
TC0100011454.hg.1	chr1	HSD11B1	hydroxysteroid (11-beta) dehydrogenase 1	NM_001206741	7.11	9.68	9.63	-2.57	-2.52
TC1700011032.hg.1	chr17	GNGT2	guanine nucleotide binding protein (G protein), gamma transd	NM_001198754	6.36	8.67	8.48	-2.30	-2.12
TC0600012293.hg.1	chr6	MB21D1	Mab-21 domain containing 1	NM_138441	4.83	6.90	6.85	-2.07	-2.02
TC0700012906.hg.1	chr7	ARHGEF35	Rho guanine nucleotide exchange factor 35	NM_001003702	5.03	7.75	7.00	-2.72	-1.96
TC1000007462.hg.1	chr10	ZNF22	zinc finger protein 22	NM_006963	6.31	8.57	8.25	-2.26	-1.94
TC0900010975.hg.1	chr9	GABBR2	gamma-aminobutyric acid (GABA) B receptor, 2	NM_005458	5.31	7.18	7.18	-1.87	-1.87
TC1000012497.hg.1	chr10	BORCS7-ASM	BORCS7-ASMT readthrough (NMD candidate)	NR_037644	4.16	5.65	6.02	-1.49	-1.85
TC1100012133.hg.1	chr11	MMP3	matrix metallopeptidase 3	NM_002422	6.55	8.50	8.40	-1.95	-1.85
TC0400012820.hg.1	chr4	FGF5	fibroblast growth factor 5	NM_001291812	6.51	8.52	8.32	-2.01	-1.81
TC0200008347.hg.1	chr2	TEX37	testis expressed 37	NM_152670	6.15	7.88	7.92	-1.73	-1.77
TC0X00011001.hg.1	chrX	MAGEC2	MAGE family member C2	NM_016249	6.17	7.99	7.93	-1.82	-1.76
TC1900010833.hg.1	chr19	PSG5	pregnancy specific beta-1-glycoprotein 5	NM_001130014	6.43	7.90	8.18	-1.47	-1.75
TC1700008635.hg.1	chr17	RGS9	regulator of G-protein signaling 9	NM_001081955	5.15	6.88	6.89	-1.74	-1.75
TC1000012496.hg.1	chr10	AS3MT	arsenite methyltransferase	NM_020682	4.25	5.67	6.00	-1.42	-1.75
TC1800007508.hg.1	chr18	TNFRSF11A	Transcript Identified by AceView, Entrez Gene ID(s) 8792	TNFRSF11A.dAug10-u	5.10	6.50	6.83	-1.40	-1.73
TC0100015598.hg.1	chr1	TXNIP	thioredoxin interacting protein	NM_006472	6.85	9.01	8.54	-2.16	-1.68
TC1800008952.hg.1	chr18	DSEL	dermatan sulfate epimerase-like	NM_032160	6.04	8.09	7.70	-2.05	-1.65
TC2000007016.hg.1	chr20	GINS1	GINS complex subunit 1 (Psf1 homolog)	NM_021067	7.88	9.25	9.45	-1.37	-1.56
TC1200011752.hg.1	chr12	GLT8D2	glycosyltransferase 8 domain containing 2	NM_031302	6.37	7.92	7.91	-1.55	-1.54
TC0X00009672.hg.1	chrX	PAGE1	P antigen family, member 1 (prostate associated)	NM_003785	7.29	8.91	8.81	-1.62	-1.52
TC1200011845.hg.1	chr12	SELPLG	selectin P ligand	NM_001206609	5.44	7.31	6.93	-1.87	-1.48
TC1200012719.hg.1	chr12	WDR66	WD repeat domain 66	NM_001178003	7.61	9.25	9.08	-1.65	-1.48
TC2000006442.hg.1	chr20	NRSN2	neurensin 2	NM_024958	6.81	8.32	8.28	-1.51	-1.47
TC0800006623.hg.1	chr8	DEFB103A	defensin, beta 103A	ENST00000314357	5.73	8.02	7.20	-2.29	-1.47
TC2200006540.hg.1	chr22	USP18	ubiquitin specific peptidase 18	NM_017414	6.19	7.31	7.64	-1.12	-1.45
TC0X00008767.hg.1	chrX	MAGEA1	MAGE family member A1	NM_004988	7.87	9.21	9.28	-1.34	-1.41
TC2200008036.hg.1	chr22	USP41	ubiquitin specific peptidase 41	OTTHUMT0000007583	6.67	8.35	8.05	-1.68	-1.38
TC0900011203.hg.1	chr9	PTGR1	prostaglandin reductase 1	NM_001146108	6.39	8.15	7.75	-1.75	-1.36
TC0600007788.hg.1	chr6	SCUBE3	signal peptide, CUB domain, EGF-like 3	NM_001303136	6.23	7.34	7.59	-1.11	-1.36
TC0500013372.hg.1	chr5	ECSCR	endothelial cell surface expressed chemotaxis and apoptosis r	NM_001077693	8.51	10.07	9.86	-1.56	-1.35
TC0800011148.hg.1	chr8	CCNE2	cyclin E2	NM_057749	4.80	6.06	6.14	-1.26	-1.34
TC0900010971.hg.1	chr9	TBC1D2	TBC1 domain family, member 2	NM_001267571	9.52	10.85	10.85	-1.34	-1.33
TC0X00008860.hg.1	chrX	F8A2	coagulation factor VIII-associated 2	NM_001007523	7.92	9.17	9.25	-1.25	-1.33
TC0900009711.hg.1	chr9	IFNE	interferon, epsilon	NM_176891	8.06	9.42	9.38	-1.37	-1.32
TC0500009033.hg.1	chr5	SPINK6	serine peptidase inhibitor, Kazal type 6	NM_001195290	4.40	5.63	5.71	-1.23	-1.31
TC0700008292.hg.1	chr7	STEAP1	six transmembrane epithelial antigen of the prostate 1	NM_012449	9.16	10.53	10.46	-1.37	-1.30
TC0700010719.hg.1	chr7	TBX20	T-box 20	NM_001077653	5.07	5.90	6.36	-0.83	-1.29
TC1200010601.hg.1	chr12	FKBP11	FK506 binding protein 11	NM_001143781	7.53	9.01	8.81	-1.48	-1.29
TC0100016018.hg.1	chr1	CRABP2	cellular retinoic acid binding protein 2	NM_001199723	5.73	7.34	7.01	-1.60	-1.28
TC0X00011244.hg.1	chrX	F8A3	coagulation factor VIII-associated 3	ENST00000622749	7.67	8.72	8.93	-1.05	-1.26
TC0800011646.hg.1	chr8	HAS2	hyaluronan synthase 2	NM_005328	6.89	8.41	8.15	-1.52	-1.25
TC0X00010670.hg.1	chrX	TMEM255A	transmembrane protein 255A	NM_001104544	5.65	7.13	6.90	-1.48	-1.25
TC0500012210.hg.1	chr5	TMEM173	transmembrane protein 173	NM_001301738	9.44	10.40	10.68	-0.96	-1.25
TC1200011460.hg.1	chr12	KITLG	KIT ligand	NM_000899	5.60	7.39	6.84	-1.80	-1.25
TC1500010184.hg.1	chr15	BCL2A1	BCL2-related protein A1	NM_001114735	8.72	10.28	9.96	-1.56	-1.24
TC1000010589.hg.1	chr10	VSTM4	V-set and transmembrane domain containing 4	NM_001031746	8.14	9.38	9.37	-1.24	-1.24
TC0600010825.hg.1	chr6	ADTRP	androgen-dependent TFPI-regulating protein	NM_001143948	7.11	8.30	8.33	-1.19	-1.22
TC0400010048.hg.1	chr4	ZNF518B	zinc finger protein 518B	NM_053042	6.20	6.70	7.42	-0.50	-1.22
TC2100006854.hg.1	chr21	MAP3K7CL	MAP3K7 C-terminal like	NM_001286617	4.39	5.81	5.60	-1.42	-1.21
TC0200010421.hg.1	chr2	AOX1	aldehyde oxidase 1	NM_001159	8.83	10.01	10.03	-1.18	-1.21
TC1900010479.hg.1	chr19	DMKN	dermokine	NM_001035516	9.39	10.52	10.60	-1.12	-1.20
TC2200008008.hg.1	chr22	ARVCF	Zhang2013 ALT_ACCEPTOR, ALT_DONOR, coding, INTERNAL, ir	hsa_circ_0092330	6.32	6.51	7.52	-0.19	-1.20
TC0100017258.hg.1	chr1	BATF3	basic leucine zipper transcription factor, ATF-like 3	NM_018664	8.05	9.44	9.24	-1.39	-1.19
TC1700012231.hg.1	chr17	TMEM98	transmembrane protein 98	NM_001033504	8.04	9.41	9.22	-1.37	-1.19
TC0200013922.hg.1	chr2	IL36B	interleukin 36, beta	NM_014438	5.10	6.33	6.28	-1.23	-1.18
TC1500008038.hg.1	chr15	ARNT2	aryl-hydrocarbon receptor nuclear translocator 2	NM_014862	7.56	8.48	8.73	-0.92	-1.17
TC1500010082.hg.1	chr15	CSPG4	chondroitin sulfate proteoglycan 4	NM_001897	7.14	8.29	8.31	-1.16	-1.17
TC0500008233.hg.1	chr5	C5orf30	chromosome 5 open reading frame 30	NM_033211	6.77	8.18	7.94	-1.42	-1.17
TC0400007933.hg.1	chr4	ANXA3	annexin A3	NM_005139	8.28	9.45	9.45	-1.17	-1.17
TC1700011435.hg.1	chr17	ICAM2	intercellular adhesion molecule 2	NM_000873	6.13	7.51	7.30	-1.38	-1.17
TC1300010011.hg.1	chr13	LINC00452	long intergenic non-protein coding RNA 452	NM_001278674	8.57	9.27	9.73	-0.70	-1.16
TC1100007216.hg.1	chr11	PRRG4	proline rich Gla (G-carboxyglutamic acid) 4 (transmembrane)	NM_024081	6.44	7.77	7.60	-1.33	-1.16
TC1700011774.hg.1	chr17	TRIM47	tripartite motif containing 47	NM_033452	8.34	9.24	9.48	-0.90	-1.14
TC0700010443.hg.1	chr7	STEAP1B	STEAP family member 1B	NM_001164460	6.95	8.44	8.09	-1.49	-1.14
TC0100008797.hg.1	chr1	AK5	adenylate kinase 5	NM_012093	6.84	7.99	7.97	-1.16	-1.13
TC2200007615.hg.1	chr22	RIBC2	RIB43A domain with coiled-coils 2	NM_015653	6.22	7.09	7.34	-0.86	-1.12
TC0500012525.hg.1	chr5	RP11-54C4.3	uncharacterized protein LOC100652758 [Source:RefSeq pepti	NM_001278082	6.13	6.91	7.25	-0.78	-1.12
TC1200007560.hg.1	chr12	DNAJC22	DnaJ (Hsp40) homolog, subfamily C, member 22	NM_001304944	7.31	8.60	8.42	-1.28	-1.11
TC0X00011339.hg.1	chrX	ARSE	arylsulfatase E (chondrodysplasia punctata 1)	NM_000047	6.78	7.98	7.88	-1.20	-1.10
TC0100016218.hg.1	chr1	RGS5	regulator of G-protein signaling 5	NM_001195303	5.63	6.34	6.72	-0.71	-1.09
TC0900007115.hg.1	chr9	GLIPR2	GLI pathogenesis-related 2	NM_001287010	7.12	7.84	8.21	-0.72	-1.09
TC0100018238.hg.1	chr1	DNAJB4	DnaJ (Hsp40) homolog, subfamily B, member 4	NM_007034	7.62	8.95	8.70	-1.34	-1.09
TC0800008028.hg.1	chr8	PKIA	protein kinase (cAMP-dependent, catalytic) inhibitor alpha	NM_006823	7.77	9.04	8.84	-1.27	-1.08
TC0500010822.hg.1	chr5	ACTBL2	actin, beta-like 2	NM_001017992	7.07	8.25	8.14	-1.18	-1.08
TC1000012536.hg.1	chr10	MPP7	membrane protein, palmitoylated 7	NM_173496	5.88	6.35	6.95	-0.47	-1.07
TC0X00008718.hg.1	chrX	MTMR1	myotubularin related protein 1	NM_001306144	4.84	5.94	5.91	-1.10	-1.07
TC1000012428.hg.1	chr10	AKR1C3	aldo-keto reductase family 1, member C3	NM_001253908	7.74	8.83	8.81	-1.09	-1.06
TC1100009169.hg.1	chr11	TAGLN	transgelin	NM_001001522	8.59	9.73	9.65	-1.14	-1.06
TC0100014685.hg.1	chr1	ADGRL4	adhesion G protein-coupled receptor L4	NM_022159	9.72	10.98	10.78	-1.26	-1.06
TC0600013007.hg.1	chr6	FAM184A	family with sequence similarity 184, member A	NM_001100411	4.52	5.13	5.57	-0.61	-1.05
TC1800007506.hg.1	chr18	TNFRSF11A	tumor necrosis factor receptor superfamily, member 11a, NFK	NM_001270949	6.29	7.15	7.34	-0.86	-1.05
TC2100006627.hg.1	chr21	MIR99AHG	mir-99a-let-7c cluster host gene	NR_027790	7.16	8.52	8.21	-1.35	-1.05
TC1700008920.hg.1	chr17	ITGB4	integrin beta 4	NM_000213	4.72	4.99	5.77	-0.27	-1.04
TC1500006786.hg.1	chr15	SCG5	secretogranin V	NM_001144757	8.10	9.10	9.13	-1.01	-1.03
TC2000006559.hg.1	chr20	CDC25B	cell division cycle 25B	NM_001287516	8.88	9.84	9.90	-0.96	-1.02
TC0300010557.hg.1	chr3	NEK10	NIMA-related kinase 10	NM_001031741	4.95	7.11	5.97	-2.16	-1.02
TC1700010973.hg.1	chr17	COPZ2	coatomer protein complex subunit zeta 2	NM_016429	9.06	10.26	10.07	-1.21	-1.02
TC0700009233.hg.1	chr7	BPGM	Transcript Identified by AceView, Entrez Gene ID(s) 669	BPGM.iAug10-unsplice	4.88	6.32	5.89	-1.44	-1.01
TC1300008249.hg.1	chr13	GJB2	gap junction protein beta 2	NM_004004	7.26	8.59	8.26	-1.33	-1.01
TC0700008657.hg.1	chr7	LRRC17	leucine rich repeat containing 17	NM_001031692	6.10	6.87	7.11	-0.77	-1.01
TC0500008470.hg.1	chr5	SNCAIP	synuclein alpha interacting protein	NM_001242935	5.25	6.32	6.25	-1.07	-1.00

Supplementary Table 2B: Genes upregulated in L-82 after stimulation with BEZ-235 (300 nM)

transcript_cluster_id	seqname	symbol	description	gb	L82_c	L82_BEZ_3h	L82_BEZ_6h	log2FC_L82_c_vs_3h	log2FC_L82_c_vs_6h
TC1100013135.hg.1	chr11	OR51B5	olfactory receptor, family 51, subfamily B, membe	NM_001005567	6.95	7.93	9.98	-0.98	-3.03
TC2000007830.hg.1	chr20	TFAP2C	transcription factor AP-2 gamma (activating enha	NM_003222	6.71	6.77	9.30	-0.05	-2.58
TC2200007150.hg.1	chr22	TIMP3	TIMP metallopeptidase inhibitor 3	NM_000362	8.02	9.14	10.60	-1.12	-2.57
TC0700006844.hg.1	chr7	ITGB8	integrin beta 8	NM_002214	6.70	7.60	9.01	-0.90	-2.31
TC1100007400.hg.1	chr11	TSPAN18	tetraspanin 18	NM_130783	6.83	7.18	9.06	-0.35	-2.23
TC2100006659.hg.1	chr21	CXADR	coxsackie virus and adenovirus receptor	NM_001207063	5.07	4.91	7.24	0.16	-2.17
TC2000009317.hg.1	chr20	SULF2	sulfatase 2	NM_001161841	6.87	7.38	8.98	-0.51	-2.11
TC0X00008674.hg.1	chrX	AFF2	AF4/FMR2 family, member 2	NM_001169122	5.47	5.28	7.52	0.19	-2.06
TC1100009924.hg.1	chr11	OR51B4	olfactory receptor, family 51, subfamily B, membe	NM_033179	6.45	7.42	8.48	-0.96	-2.03
TC0300009773.hg.1	chr3	FETUB	fetuin B	NM_001308077	6.08	7.74	8.09	-1.66	-2.01
TC0600008972.hg.1	chr6	PRDM1	PR domain containing 1, with ZNF domain	NM_001198	6.27	6.67	8.27	-0.41	-2.00
TC0100011391.hg.1	chr1	IL24	interleukin 24	NM_001185156	8.71	8.29	10.69	0.42	-1.98
TC1900009584.hg.1	chr19	ZNF560	zinc finger protein 560	NM_152476	5.25	5.44	7.22	-0.19	-1.96
TC1200010341.hg.1	chr12	PKP2	plakophilin 2	NM_001005242	7.00	8.42	8.97	-1.42	-1.96
TC1900009576.hg.1	chr19	ZNF699	zinc finger protein 699	NM_198535	5.05	5.43	6.97	-0.38	-1.92
TC1800007340.hg.1	chr18	DCC	DCC netrin 1 receptor	NM_005215	4.58	4.61	6.44	-0.04	-1.87
TC0600006869.hg.1	chr6	DSP	desmoplakin	NM_001008844	5.42	5.97	7.28	-0.55	-1.87
TC0100015194.hg.1	chr1	SORT1	sortilin 1	NM_001205228	4.87	5.18	6.73	-0.31	-1.86
TC1000008193.hg.1	chr10	PPIF	peptidylprolyl isomerase F	NM_005729	7.31	8.42	9.16	-1.12	-1.86
TC1600010561.hg.1	chr16	CDH11	cadherin 11, type 2, OB-cadherin (osteoblast)	NM_001308392	6.86	8.03	8.70	-1.17	-1.84
TC1000006768.hg.1	chr10	CELF2	Transcript Identified by AceView, Entrez Gene ID	CELF2.vIAug10-unsp	4.63	4.44	6.44	0.19	-1.81
TC0900011305.hg.1	chr9	TNC	tenascin C	NM_002160	7.11	9.44	8.92	-2.33	-1.81
TC0300013954.hg.1	chr3	ZNF385D	zinc finger protein 385D	NM_024697	5.69	6.87	7.49	-1.19	-1.80
TC1700009694.hg.1	chr17	MYH10	myosin, heavy chain 10, non-muscle	NM_001256012	8.92	9.22	10.69	-0.31	-1.77
TC0Y00006648.hg.1	chrY	VCY1B	variable charge, Y-linked 1B	ENST00000250823	6.96	7.13	8.72	-0.18	-1.76
TC1400010012.hg.1	chr14	LGMN	legumain	NM_001008530	7.41	9.33	9.17	-1.91	-1.76
TC0Y00007078.hg.1	chrY	VCY	variable charge, Y-linked	NM_004679	7.19	7.43	8.94	-0.25	-1.75
TC1900009443.hg.1	chr19	C3	complement component 3	NM_000064	5.77	6.19	7.51	-0.42	-1.74
TC0X00010643.hg.1	chrX	SEPT6	septin 6	NM_015129	7.83	8.61	9.56	-0.78	-1.73
TC0600008760.hg.1	chr6	PM20D2	peptidase M20 domain containing 2	NM_001010853	8.27	8.78	9.98	-0.51	-1.71
TC0400010141.hg.1	chr4	LDB2	LIM domain binding 2	NM_001130834	5.70	5.63	7.41	0.08	-1.71
TC0600011945.hg.1	chr6	RCAN2	regulator of calcineurin 2	NM_001251973	5.78	6.55	7.48	-0.76	-1.70
TC0100017018.hg.1	chr1	ETNK2	ethanolamine kinase 2	NM_001297760	7.27	7.64	8.96	-0.36	-1.68
TC1000011485.hg.1	chr10	PDLIM1	PDZ and LIM domain 1	NM_020992	7.54	8.60	9.19	-1.06	-1.65
TC0X00009672.hg.1	chrX	PAGE1	P antigen family, member 1 (prostate associated)	NM_003785	5.63	6.87	7.28	-1.24	-1.65
TC0400009904.hg.1	chr4	PPP2R2C	protein phosphatase 2, regulatory subunit B, gam	NM_001206994	5.77	6.04	7.38	-0.27	-1.62
TC1100006988.hg.1	chr11	MRGPRX4	MAS-related GPR, member X4	NM_054032	6.74	6.70	8.35	0.05	-1.61
TC0Y00007074.hg.1	chrY	UTY	ubiquitously transcribed tetratricopeptide repeat c	NM_001258249	5.30	6.25	6.90	-0.95	-1.60
TC2000007157.hg.1	chr20	CBFA2T2	core-binding factor, runt domain, alpha subunit 2;	NM_001032999	6.17	6.47	7.75	-0.30	-1.58
TC0400007500.hg.1	chr4	SPATA18	spermatogenesis associated 18	NM_001297608	5.50	5.88	7.08	-0.38	-1.58
TC0100006650.hg.1	chr1	AJAP1	adherens junctions associated protein 1	NM_001042478	7.95	8.50	9.50	-0.55	-1.56
TC0900010886.hg.1	chr9	PTCH1	patched 1	NM_000264	5.73	5.37	7.28	0.36	-1.55
TC0X00010459.hg.1	chrX	MORC4	MORC family CW-type zinc finger 4	NM_001085354	5.88	6.77	7.43	-0.89	-1.55
TC0700007034.hg.1	chr7	CREB5	cAMP responsive element binding protein 5	NM_001011666	6.92	7.93	8.46	-1.02	-1.54
TC0200008894.hg.1	chr2	MERTK	MER proto-oncogene, tyrosine kinase	NM_006343	4.26	4.95	5.80	-0.68	-1.54
TC1200012748.hg.1	chr12	CD163L1	CD163 molecule-like 1	NM_001297650	6.56	7.33	8.08	-0.77	-1.53
TC1100010505.hg.1	chr11	ABTB2	ankyrin repeat and BTB (POZ) domain containing	NM_145804	6.74	7.30	8.23	-0.56	-1.49
TC0500013039.hg.1	chr5	ADAMTS2	ADAM metallopeptidase with thrombospondin typ	NM_014244	8.31	8.30	9.79	0.01	-1.48
TC0500007258.hg.1	chr5	GHR	growth hormone receptor	NM_000163	4.59	5.35	6.06	-0.77	-1.47
TC0600006967.hg.1	chr6	EDN1	endothelin 1	NM_001168319	7.18	6.85	8.64	0.32	-1.46
TC0600012061.hg.1	chr6	ICK	intestinal cell (MAK-like) kinase	NM_014920	8.15	8.20	9.58	-0.05	-1.44
TC0500010540.hg.1	chr5	LIFR	leukemia inhibitory factor receptor alpha	NM_001127671	4.49	5.11	5.91	-0.62	-1.42
TC1100013045.hg.1	chr11	SIPA1	signal-induced proliferation-associated 1	NM_006747	7.08	7.21	8.49	-0.13	-1.41
TC0300012814.hg.1	chr3	GPR87	G protein-coupled receptor 87	NM_023915	5.11	6.36	6.51	-1.25	-1.41
TC0800011611.hg.1	chr8	TNFRSF11B	tumor necrosis factor receptor superfamily, memt	NM_002546	5.33	5.95	6.73	-0.62	-1.41
TC0X00010126.hg.1	chrX	ATRX	alpha thalassemia/mental retardation syndrome X	NM_000489	5.28	6.23	6.68	-0.95	-1.40
TC1700008133.hg.1	chr17	KPNB1	karyopherin (importin) beta 1	NM_001276453	10.13	11.27	11.50	-1.14	-1.37
TC1600008301.hg.1	chr16	CALB2	calbindin 2	NM_001740	8.48	8.74	9.85	-0.27	-1.37
TC2200006833.hg.1	chr22	BCR	breakpoint cluster region	NM_004327	5.36	6.05	6.73	-0.69	-1.37
TC1900008967.hg.1	chr19	ZNF470	zinc finger protein 470	NM_001001668	5.88	5.74	7.23	0.14	-1.36
TC1400007774.hg.1	chr14	NRXN3	neurexin 3	NM_001105250	5.65	6.59	7.00	-0.94	-1.35
TC1700010230.hg.1	chr17	TIAF1	TGFB1-induced anti-apoptotic factor 1	NM_004740	7.57	7.50	8.92	0.07	-1.35
TC0X00006567.hg.1	chrX	VCX3B	variable charge, X-linked 3B	NM_001001888	7.03	6.84	8.37	0.18	-1.34
TC1000012117.hg.1	chr10	CHST15	carbohydrate (N-acetylgalactosamine 4-sulfate 6-	NM_001270764	7.55	8.41	8.89	-0.85	-1.34
TC0100011520.hg.1	chr1	PPP2R5A	protein phosphatase 2, regulatory subunit B, alpha	NM_001199756	6.36	6.89	7.67	-0.53	-1.31
TC0200009700.hg.1	chr2	GALNT13	polypeptide N-acetylgalactosaminyltransferase 13	NM_001301627	4.96	4.83	6.26	0.13	-1.30
TC1100013152.hg.1	chr11	SAA2	serum amyloid A2	NM_001127380	5.25	5.29	6.55	-0.04	-1.30
TC1800006937.hg.1	chr18	TAF4B	TAF4b RNA polymerase II, TATA box binding pro	NM_001293725	6.11	6.59	7.38	-0.48	-1.28
TC1400007495.hg.1	chr14	PLEKHH1	pleckstrin homology domain containing, family H	NM_020715	7.22	7.47	8.49	-0.25	-1.27
TC0900006559.hg.1	chr9	CD274	CD274 molecule	NM_001267706	7.81	9.04	9.08	-1.23	-1.27
TC1900009439.hg.1	chr19	CD70	CD70 molecule	NM_001252	7.70	9.38	8.96	-1.68	-1.26
TC1400008767.hg.1	chr14	STXBP6	syntaxin binding protein 6 (amisyn)	NM_001304476	8.66	9.02	9.92	-0.35	-1.26
TC0X00009025.hg.1	chrX	ANOS1	anosmin 1	NM_000216	9.50	10.21	10.75	-0.71	-1.25
TC0900010390.hg.1	chr9	ABHD17B	abhydrolase domain containing 17B	NM_001025780	7.25	7.73	8.48	-0.48	-1.23
TC0500007050.hg.1	chr5	PDZD2	PDZ domain containing 2	NM_178140	6.10	5.93	7.33	0.17	-1.23
TC0X00006558.hg.1	chrX	VCX	variable charge, X-linked	NM_013452	7.36	7.18	8.58	0.18	-1.22
TC0500008175.hg.1	chr5	RGMB	repulsive guidance molecule family member b	NM_001012761	6.96	8.27	8.18	-1.31	-1.22
TC1100012722.hg.1	chr11	CDON	cell adhesion associated, oncogene regulated	NM_001243597	5.25	5.27	6.47	-0.02	-1.22
TC0100009241.hg.1	chr1	S1PR1	sphingosine-1-phosphate receptor 1	NM_001400	8.31	7.83	9.53	0.48	-1.22
TC0X00007493.hg.1	chrX	AR	androgen receptor	NM_000044	6.57	7.37	7.79	-0.80	-1.22
TC0X00009022.hg.1	chrX	VCX2	variable charge, X-linked 2	NM_016378	7.23	7.03	8.44	0.20	-1.21
TC0300009353.hg.1	chr3	PPM1L	protein phosphatase, Mg2+/Mn2+ dependent, 1L	NM_139245	5.17	5.53	6.38	-0.36	-1.21
TC0300008324.hg.1	chr3	PHLDB2	pleckstrin homology-like domain, family B, memb	NM_001134437	8.52	9.29	9.72	-0.77	-1.20
TC1700010358.hg.1	chr17	MYO1D	myosin ID	NM_001303279	6.27	7.20	7.47	-0.93	-1.20
TC1100010207.hg.1	chr11	SOX6	SRY box 6	NM_001145811	4.59	4.51	5.79	0.08	-1.20
TC0900007680.hg.1	chr9	TLE4	transducin-like enhancer of split 4	NM_001282748	6.07	6.71	7.25	-0.64	-1.18
TC0X00008040.hg.1	chrX	MUM1L1	melanoma associated antigen (mutated) 1-like 1	NM_001171020	5.73	5.94	6.91	-0.21	-1.18
TC2000007973.hg.1	chr20	CDH4	cadherin 4, type 1, R-cadherin (retinal)	NM_001252339	6.01	6.08	7.18	-0.07	-1.18
TC1900008141.hg.1	chr19	CYP2S1	cytochrome P450, family 2, subfamily S, polypept	NM_030622	8.17	8.39	9.34	-0.22	-1.17
TC0800009905.hg.1	chr8	NEFL	neurofilament, light polypeptide	NM_006158	6.99	7.47	8.16	-0.48	-1.17
TC1100011234.hg.1	chr11	LTBP3	latent transforming growth factor beta binding pro	NM_001130144	9.68	10.74	10.85	-1.05	-1.17
TC1900009867.hg.1	chr19	AKAP8L	A kinase (PRKA) anchor protein 8-like	NM_001291478	9.06	9.55	10.23	-0.49	-1.17
TC2100006974.hg.1	chr21	SON	SON DNA binding protein	NM_001291411	8.54	9.30	9.70	-0.76	-1.16
TC0500007138.hg.1	chr5	IL7R	interleukin 7 receptor	NM_002185	5.81	5.75	6.97	0.05	-1.16
TC0300011264.hg.1	chr3	IL17RD	interleukin 17 receptor D	NM_017563	5.20	5.70	6.36	-0.50	-1.16
TC1700008350.hg.1	chr17	NOG	noggin	NM_005450	7.44	8.42	8.60	-0.98	-1.16

TC1300008225.hg.1	chr13	PSPC1	paraspeckle component 1	NM_001042414	8.73	9.55	9.88	-0.82	-1.16
TC0800012362.hg.1	chr8	KHDRBS3	KH domain containing, RNA binding, signal transd	NM_006558	6.81	6.92	7.96	-0.11	-1.15
TC1100013133.hg.1	chr11	HBG2	hemoglobin, gamma G	NM_000184	5.67	6.05	6.82	-0.38	-1.15
TC0100007645.hg.1	chr1	TINAGL1	tubulointerstitial nephritis antigen-like 1	NM_001204414	7.00	7.18	8.15	-0.18	-1.15
TC1100006760.hg.1	chr11	PPFIBP2	PTPRF interacting protein, binding protein 2 (lipri	NM_001256568	5.05	5.20	6.20	-0.15	-1.15
TC2000009023.hg.1	chr20	SAMHD1	SAM domain and HD domain 1	NM_015474	6.04	6.62	7.19	-0.58	-1.15
TC0300013255.hg.1	chr3	ZMAT3	zinc finger, matrin-type 3	NM_022470	7.64	8.42	8.78	-0.79	-1.15
TC0800008715.hg.1	chr8	ZHX2	zinc fingers and homeoboxes 2	NM_014943	6.50	7.32	7.64	-0.82	-1.14
TC0X00009000.hg.1	chrX	VCX3A	variable charge, X-linked 3A	NM_016379	7.08	7.03	8.22	0.05	-1.14
TC1200012424.hg.1	chr12	TMEM132D	transmembrane protein 132D	NM_133448	5.45	4.96	6.59	0.49	-1.14
TC0100007638.hg.1	chr1	SERINC2	serine incorporator 2	NM_001199037	10.30	10.95	11.43	-0.65	-1.13
TC0400009543.hg.1	chr4	TLR3	toll-like receptor 3	NM_003265	5.32	6.55	6.44	-1.24	-1.12
TC0900007618.hg.1	chr9	PCSK5	proprotein convertase subtilisin/kexin type 5	NM_001190482	5.65	6.05	6.78	-0.40	-1.12
TC0200012997.hg.1	chr2	TGFA	transforming growth factor alpha	NM_001099691	9.21	9.35	10.33	-0.14	-1.12
TC1200008081.hg.1	chr12	DYRK2	dual specificity tyrosine-(Y)-phosphorylation regul	NM_003583	5.85	6.23	6.96	-0.38	-1.11
TC0200013298.hg.1	chr2	ST3GAL5	ST3 beta-galactoside alpha-2,3-sialyltransferase 5	NM_001042437	5.69	5.17	6.79	0.52	-1.10
TC2000009058.hg.1	chr20	TGM2	transglutaminase 2	NM_004613	10.49	11.00	11.58	-0.51	-1.09
TC0300007617.hg.1	chr3	KCTD6	potassium channel tetramerization domain contai	NM_001128214	4.87	5.37	5.96	-0.50	-1.09
TC0Y00006495.hg.1	chrY	TGIF2LY	TGFB-induced factor homeobox 2-like, Y-linked	NM_139214	4.42	4.63	5.51	-0.22	-1.09
TC0100015991.hg.1	chr1	SMG5	SMG5 nonsense mediated mRNA decay factor	NM_015327	8.52	9.04	9.61	-0.51	-1.09
TC1900006864.hg.1	chr19	MAP2K7	mitogen-activated protein kinase kinase 7	NM_001297555	6.75	7.23	7.83	-0.48	-1.09
TC1100007220.hg.1	chr11	DEPDC7	DEP domain containing 7	NM_001077242	5.38	5.39	6.46	-0.01	-1.08
TC0900011623.hg.1	chr9	PTGES2	prostaglandin E synthase 2	NM_001256335	6.96	7.77	8.04	-0.80	-1.08
TC1500006925.hg.1	chr15	THBS1	thrombospondin 1	NM_003246	11.87	12.06	12.95	-0.19	-1.08
TC1900007382.hg.1	chr19	PGPEP1	pyroglutamyl-peptidase I	NM_001300927	8.14	8.66	9.22	-0.52	-1.07
TC2000007343.hg.1	chr20	DHX35	DEAH (Asp-Glu-Ala-His) box polypeptide 35	NM_001190809	6.34	6.74	7.41	-0.40	-1.07
TC0600014318.hg.1	chr6	FAM46A	family with sequence similarity 46, member A	NM_017633	7.49	7.82	8.55	-0.34	-1.06
TC1100012574.hg.1	chr11	SORL1	Memczak2013 ANTISENSE, CDS, coding, INTER	hsa_circ_0000366	6.70	7.20	7.76	-0.50	-1.06
TC1100013163.hg.1	chr11	APIP	APAF1 interacting protein	NM_015957	7.54	8.58	8.60	-1.04	-1.06
TC1900009558.hg.1	chr19	ZNF558	zinc finger protein 558	NM_001304350	6.06	6.41	7.12	-0.35	-1.06
TC2000008104.hg.1	chr20	PRPF6	pre-mRNA processing factor 6	NM_012469	7.90	8.32	8.95	-0.42	-1.05
TC0800012363.hg.1	chr8	KHDRBS3	KH domain containing, RNA binding, signal transd	ENST00000522578	5.78	5.99	6.83	-0.21	-1.05
TC1200011812.hg.1	chr12	CRY1	cryptochrome circadian clock 1	NM_004075	6.14	6.56	7.19	-0.42	-1.05
TC1100009929.hg.1	chr11	OR51I1	olfactory receptor, family 51, subfamily I, member	NM_001005288	5.84	5.94	6.89	-0.10	-1.05
TC1100009926.hg.1	chr11	OR51B2	olfactory receptor, family 51, subfamily B, membe	NM_033180	5.09	5.42	6.14	-0.33	-1.04
TC0400009765.hg.1	chr4	MXD4	MAX dimerization protein 4	NM_006454	7.73	8.45	8.78	-0.72	-1.04
TC0200008680.hg.1	chr2	SLC9A2	solute carrier family 9, subfamily A (NHE2, cation	NM_003048	4.39	4.09	5.43	0.30	-1.04
TC0900009785.hg.1	chr9	LINGO2	leucine rich repeat and Ig domain containing 2	NM_001258282	4.40	4.58	5.44	-0.19	-1.04
TC0100018443.hg.1	chr1	PLPP3	phospholipid phosphatase 3	NM_003713	8.56	7.29	9.59	1.27	-1.04
TC0300008853.hg.1	chr3	TMEM108	transmembrane protein 108	NM_001136469	6.39	6.25	7.42	0.13	-1.03
TC1700008209.hg.1	chr17	NGFR	nerve growth factor receptor	NM_002507	6.71	6.68	7.74	0.02	-1.03
TC1100012478.hg.1	chr11	CD3D	CD3d molecule, delta (CD3-TCR complex)	NM_000732	5.23	5.91	6.26	-0.68	-1.03
TC1400010774.hg.1	chr14	FOXN3	forkhead box N3	NM_001085471	6.12	6.58	7.14	-0.46	-1.03
TC0400010242.hg.1	chr4	PPARGC1A	peroxisome proliferator-activated receptor gamma	NM_013261	7.69	7.64	8.72	0.05	-1.02
TC2000009646.hg.1	chr20	CTSZ	cathepsin Z	NM_001336	9.79	10.31	10.81	-0.51	-1.02
TC1200012161.hg.1	chr12	TRIAP1	TP53 regulated inhibitor of apoptosis 1	NM_016399	8.40	9.23	9.43	-0.83	-1.02
TC0500007452.hg.1	chr5	MAP3K1	mitogen-activated protein kinase kinase kinase 1,	NM_005921	5.37	5.46	6.39	-0.09	-1.02
TC0X00007053.hg.1	chrX	MAOA	monoamine oxidase A	NM_000240	6.80	7.16	7.82	-0.36	-1.02
TC0800010002.hg.1	chr8	DUSP4	dual specificity phosphatase 4	NM_001394	10.66	10.90	11.68	-0.24	-1.02
TC0600007067.hg.1	chr6	GMPR	guanosine monophosphate reductase	NM_006877	6.93	7.42	7.95	-0.49	-1.02
TC0500009610.hg.1	chr5	NSD1	nuclear receptor binding SET domain protein 1	NM_022455	7.60	7.82	8.61	-0.23	-1.02
TC0200012339.hg.1	chr2	SLC8A1	solute carrier family 8 (sodium/calcium exchange	NM_001112800	4.60	4.60	5.62	0.01	-1.02
TC0600012434.hg.1	chr6	ME1	malic enzyme 1, NADP(+)-dependent, cytosolic	NM_002395	8.55	9.62	9.56	-1.07	-1.01
TC1900006932.hg.1	chr19	ZNF559-ZNF1	ZNF559-ZNF177 readthrough	NM_001172650	5.47	5.72	6.48	-0.26	-1.01
TC1200012741.hg.1	chr12	ANO2	anoctamin 2, calcium activated chloride channel	NM_001278596	4.84	4.89	5.86	-0.05	-1.01
TC1900009518.hg.1	chr19	FBN3	Zhang2013 ALT_ACCEPTOR, ALT_DONOR, cod	hsa_circ_0092332	5.13	5.36	6.14	-0.23	-1.01
TC0300008561.hg.1	chr3	PARP14	poly(ADP-ribose) polymerase family member 14	NM_017554	8.95	9.34	9.96	-0.38	-1.01
TC0200015093.hg.1	chr2	ZNF385B	zinc finger protein 385B	NM_001113397	4.41	4.94	5.42	-0.53	-1.01
TC0900011192.hg.1	chr9	LPAR1	lysophosphatidic acid receptor 1	NM_001401	7.19	7.56	8.20	-0.37	-1.01
TC0300011859.hg.1	chr3	ZBTB11	zinc finger and BTB domain containing 11	NM_014415	5.66	6.29	6.66	-0.63	-1.01
TC0900009479.hg.1	chr9	RANBP6	RAN binding protein 6	NM_001243202	5.17	5.63	6.17	-0.46	-1.00
TC0200010421.hg.1	chr2	AOX1	aldehyde oxidase 1	NM_001159	7.96	9.02	8.96	-1.06	-1.00
TC0200009865.hg.1	chr2	SCN2A	sodium channel, voltage gated, type II alpha subu	NM_001040142	7.06	7.54	8.07	-0.47	-1.00

Supplementary Table 3. Sequence of primers for quantitative genomic PCR.

gene			primer
BCL2 intron 2/3	forward	NM_000633	5´- CCA GGT TGG GTC TTG ACA G - 3´
BCL2 intron 2/3	reverse	NM_000633	5´- ATG ATG CCC TTG GTC TTC TGT - 3´
BCLXL exon 2	forward	NM_138578.3	5´- CCG GGA GCT GGT GGT TG - 3´
BCLXL exon 2	reverse	NM_138578.3	5´- CCT GTT CTC TTC CAC ATC ACT - 3´
MCL1 exon 1	forward	NM_021960	5´- TGG CGT GCA GCG CAA CC - 3´
MCL1 intron 1/2	reverse	NM_021960	5´- GGA GTG AGG CCT TGG CG - 3´
LINE1	forward		5´- TGC TTT GAA TGC GTC CCA GAG -3´
LINE1	reverse		5´- AAA GCC GCT CAA CTA CAT GG - 3´

PCR conditions: 59°C, 35 cycles.
